# New Model of Macrophage Acquisition of the Lymphatic Endothelial Phenotype

**DOI:** 10.1371/journal.pone.0031794

**Published:** 2012-03-02

**Authors:** Kelly L. Hall, Lisa D. Volk-Draper, Michael J. Flister, Sophia Ran

**Affiliations:** Department of Medical Microbiology, Immunology, and Cell Biology, Southern Illinois University School of Medicine, Springfield, Illinois, United States of America; Università degli Studi di Milano, Italy

## Abstract

**Background:**

Macrophage-derived lymphatic endothelial cell progenitors (M-LECPs) contribute to new lymphatic vessel formation, but the mechanisms regulating their differentiation, recruitment, and function are poorly understood. Detailed characterization of M-LECPs is limited by low frequency *in vivo* and lack of model systems allowing in-depth molecular analyses *in vitro*. Our goal was to establish a cell culture model to characterize inflammation-induced macrophage-to-LECP differentiation under controlled conditions.

**Methodology/Principal Findings:**

Time-course analysis of diaphragms from lipopolysaccharide (LPS)-treated mice revealed rapid mobilization of bone marrow-derived and peritoneal macrophages to the proximity of lymphatic vessels followed by widespread (∼50%) incorporation of M-LECPs into the inflamed lymphatic vasculature. A differentiation shift toward the lymphatic phenotype was found in three LPS-induced subsets of activated macrophages that were positive for VEGFR-3 and many other lymphatic-specific markers. VEGFR-3 was strongly elevated in the early stage of macrophage transition to LECPs but undetectable in M-LECPs prior to vascular integration. Similar transient pattern of VEGFR-3 expression was found in RAW264.7 macrophages activated by LPS *in vitro*. Activated RAW264.7 cells co-expressed VEGF-C that induced an autocrine signaling loop as indicated by VEGFR-3 phosphorylation inhibited by a soluble receptor. LPS-activated RAW264.7 macrophages also showed a 68% overlap with endogenous CD11b^+^/VEGFR-3^+^ LECPs in the expression of lymphatic-specific genes. Moreover, when injected into LPS- but not saline-treated mice, GFP-tagged RAW264.7 cells massively infiltrated the inflamed diaphragm followed by integration into 18% of lymphatic vessels.

**Conclusions/Significance:**

We present a new model for macrophage-LECP differentiation based on LPS activation of cultured RAW264.7 cells. This system designated here as the “RAW model” mimics fundamental features of endogenous M-LECPs. Unlike native LECPs, this model is unrestricted by cell numbers, heterogeneity of population, and ability to change genetic composition for experimental purposes. As such, this model can provide a valuable tool for understanding the LECP and lymphatic biology.

## Introduction

The lymphatic system has important functions in human physiology and pathology including regulation of interstitial fluid balance [1], [2], lipid absorption [3], immunity [4], inflammation [5] and metastatic spread [6], [7]. During embryogenesis, the formation of new lymphatic vessels (i.e., lymphangiogenesis) is a highly active process. In contrast, in adults, this process is largely restricted to sites of cancer [6], [7], chronic inflammation [8]–[12], and tissue remodeling [13], [14]. The key regulatory protein that induces lymphangiogenesis is the tyrosine kinase receptor VEGFR-3 [15], [16]. This protein is highly expressed in lymphatic endothelial cells (LECs) [17], [18] and upregulated in response to inflammation [17]. The central role of VEGFR-3 in lymphangiogenesis is shown by a significant reduction in lymphatic vessel density following VEGFR-3 blockade during chronic inflammation [8], wound healing [19], and malignancy [20]. In line with this evidence, we recently showed in the mouse peritonitis model of inflammatory lymphangiogenesis that activated NF-κB upregulates VEGFR-3 on inflamed lymphatic vessels [17]. This event is crucial for lymphangiogenesis because it amplifies the responsiveness of pre-existing lymphatic vessels to VEGFR-3 ligands, VEGF-C and VEGF-D [16], [21] that can be produced by many cell sources including stromal [22], epithelial [23], malignant [6] and immune cells [8], [24], [25].

Postnatal lymphangiogenesis has long been thought to occur exclusively through sprouting of pre-existing lymphatic vessels, a process that involves proliferation and migration of fully differentiated LECs [26]. Recent reports, however, have shown that lymphangiogenesis can also be regulated by bone marrow (BM)-derived lymphatic endothelial cell progenitors (LECPs) that comprise a small fraction of LECs in newly formed lymphatic vessels [27]–[32]. The role of LECPs is based on studies demonstrating that LECPs are recruited to inflamed sites [28]–[31], [33] and integrate into activated lymphatic vessels [27]–[31]. The majority of studies have suggested that LECPs are derived from myeloid cells of the monocyte/macrophage lineage [29]–[31], [33], although other sources may include embryonic stem cells (ESC) [34] and those from mesenchymal (MSC) [35] and hematopoietic (HSC) [27] origins. Macrophage-derived LECPs (M-LECPs) have been identified by lymphatic vascular integration of cells with dual positivity for myeloid (e.g., CD11b) and lymphatic-specific markers (e.g., LYVE-1). This was first demonstrated in biopsies of gender-mismatched human renal transplants that revealed integration of donor-derived macrophage LECPs into recipient lymphatic vessels [29]. Similar findings have also been reported in animal models of corneal inflammation [30], [33], wound healing [31], cancer [28], [30], [31], and in studies using adoptive BM-transfer from GFP-transgenic mice to non-transgenic recipients [28], [31]. Importantly, GFP-labeled LECPs not only quickly integrated into the nascent vessels during the first week post-transfer [31], but also remained in the lymphatic vasculature for at least six months [28]. This suggested that adult LECPs might be involved in both induction of lymphangiogenesis and the maintenance of the newly-formed vessels.

Despite the growing body of evidence indicating the important role of LECPs in lymphangiogenesis, little is known about the LECP phenotype, mechanisms of recruitment, differentiation into mature LECs, and roles in vascular remodeling. The obstacles to gaining this information are mainly due to three reasons: 1) low (2–5%) frequency of LECP incorporation into vessels [27]–[29], 2) limits of the methods for detection of LECPs, and 3) difficulties monitoring their fate *in vivo* due to loss of myeloid markers after integration into lymphatic vasculature. These difficulties are further compounded by macrophage secretion of pro-lymphangiogenic factors (i.e., VEGF-C, -D and -A) that stimulate lymphangiogenesis directly, without integration of macrophage-derived progenitors into vasculature. Consequently, while a macrophage depletion method can be successfully used to demonstrate dependence of lymphangiogenesis on M-LECPs [28], [33], this approach does not discern between the paracrine effects of macrophage-derived lymphangiogenic factors and the autonomous roles of M-LECPs.

These challenges prompted us to search for a cell culture model that can be manipulated under controlled conditions to allow delineation of the molecular and cellular events underlying the lymphangiogenic function of adult M-LECPs. This approach has been successfully used to model blood vascular endothelial cell progenitors (BVECPs) [36] suggesting that a similar strategy can be applied to modeling macrophage-to-LECP transdifferentiation. Since M-LECPs are known to partake in inflammatory lymphangiogenesis [29]–[31], [33], we hypothesized that the lymphatic phenotype can be induced in cultured macrophages by an inflammatory stimulator such as LPS. We found that LPS treatment of RAW264.7 macrophages, a cell line that normally lacks LEC markers, induces coincident *de novo* expression of VEGFR-3 and VEGF-C leading to establishment of a novel autocrine loop. Activation of VEGFR-3 pathway prompted macrophages to express a variety of lymphatic-specific genes, including LYVE-1, c-Maf, integrin alpha9, Notch1 and podoplanin. Moreover, upon injection into LPS- but not saline-treated mice, GFP-tagged RAW264.7 macrophages (RAW-GFP) formed large clusters that first firmly adhered to lymphatic endothelium followed by integration into approximately one-fifth of the inflamed vessels. This behavior recapitulated that of endogenous M-LECPs which were found to be first massively recruited to diaphragms in LPS-treated mice followed by quick incorporation into ∼50% of the inflamed lymphatic vasculature. RT-qPCR analysis showed that LPS-activated RAW264.7 cells *in vitro* and endogenous VEGFR-3^+^ M-LECPs isolated from LPS-treated mice have a 68% overlap in expression of lymphatic-specific genes. Collectively, these findings suggest that LPS-treated macrophage RAW264.7 line recapitulates both gene expression profile and the biological behavior of M-LECPs recruited to inflammatory lymphangiogenic sites *in vivo*. We, therefore, believe that this novel model of macrophage-to-LECP differentiation can provide a unique means for delineating molecular, cellular, and systemic mechanisms of inflammatory lymphangiogenesis both *in vitro* and *in vivo*.

## Results

### LPS induces VEGFR-3 expression in several subsets of activated macrophages *in vivo*

Macrophages expressing LEC-markers that may function as LECPs have been previously detected in malignant [28], [37] and wound healing models [13], [38]. However, detailed molecular and cellular characterization of these cells has been hindered by the inability to isolate large numbers of phenotypically homogeneous LECPs due to their low frequency *in situ* [27], [28]. To overcome this obstacle, we sought to establish a cell culture model that would allow delineation of the molecular mechanisms driving macrophage differentiation into LECPs.

**Figure 1:**
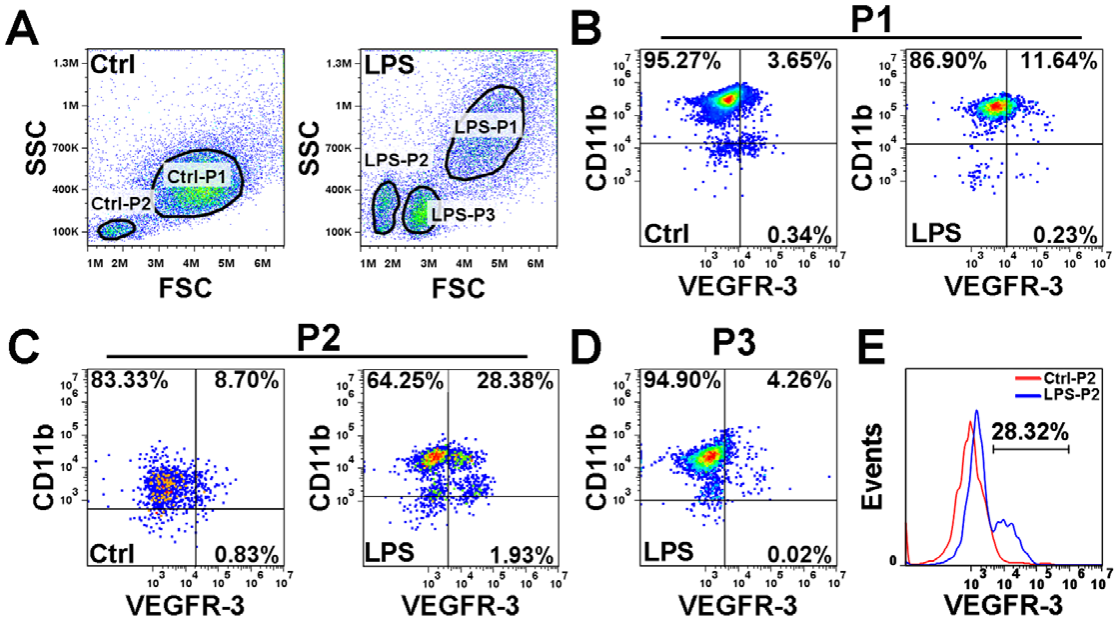
VEGFR-3 protein is elevated on several subsets of macrophage-derived LECP (M-LECP) *in vivo*. Balb/c mice were injected i.p. once with 100 ul of sterile endotoxin-free saline or LPS (50 µg) and treated for 24 hours. (**A**) Cells were isolated using magnetic beads conjugated to anti-CD11b antibody and analyzed by flow cytometry. Two major cell populations in control and three in LPS were identified and analyzed separately for dual expression of CD11b and VEGFR-3. Gating is indicated by black lines. Populations P1 (**B**) and P2 (**C**) were found in both control and LPS-activated CD11b^+^ cells. (**D**) Population P3 was present only in LPS-treated mice. (**B–D**) Expression of VEGFR-3 on LPS-activated and control CD11b^+^ isolated cells was analyzed. Numbers in each quadrant represent the percentage of positive cells for the indicated protein. Three independent experiments were performed with similar results (for each experiment: n = 5 mice per group). (**E**) Histogram of VEGFR-3 expression in population P2 showing the largest increase in VEGFR-3 protein expression in response to LPS treatment.

Towards this goal, we first characterized the endogenous LECPs recruited to an inflammatory site. This was done in a mouse peritonitis model induced by a single i.p. injection of LPS (50 µg), a method reported to induce both macrophage recruitment [39] and lymphangiogenesis in the diaphragm [17]. Resident control macrophages were obtained from the peritoneum of saline-injected mice. LPS-activated and control CD11b^+^ cells were isolated using magnetic beads conjugated to anti-CD11b antibody, and analyzed by flow cytometry for dual expression of VEGFR-3 and CD11b. These cells were also stained for the expression of the lymphatic markers LYVE-1 and podoplanin as well as myeloid markers F4/80, CD11c and Ly6C. Fig. 1A shows that resident macrophages can be split into two populations (labeled as Ctrl-P1 and Ctrl-P2) whereas after LPS-induced population can be subdivided into three subsets with distinct scatter properties (designated as LPS-P1, LPS-P2 and LPS-P3). LPS-P1 was likely comprised of mature macrophages as indicated by their large size, 92% positivity for F4/80 [40] and low expression of other markers (Fig. 1, Table 1). In contrast, the two smaller populations were largely negative for F4/80 and highly positive (70–80%) for Ly6C (Table 1). These two populations, LPS-P2 and LPS-P3, likely represent monocyte progenitor cells recruited from the bone marrow through blood vessels [41].

**Table 1:** Expression of VEGFR-3 in subsets of CD11b^+^ macrophages before and after treatment with LPS.

	Ctrl-P1†	LPS-P1	Ctrl-P2	LPS-P2	LPS-P3
**Lymphatic Markers**					
** VEGFR-3**	3.7±0.6	9.2±0.8 (<0.001)¥	7.7±0.5	26.0±0.8 (<0.001)	5.8±0.5 (n.s.)
** LYVE1**	5.6±0.5	4.3±0.9 (<0.001)	ND§	8.1±0.8 (<0.05)	3.0±0.5
** Podoplanin**	ND	9.1±0.2 (<0.01)	29.7±1.49	33.5±1.4 (0.001)	4.7±0.4 (<0.001)
**Myeloid Markers**					
** F4/80**	84.4±1.2	92.3±1.9 (n.s.)	ND	ND	3.1±0.6 (<0.05)
** CD11c**	ND	ND	32.4±3.4	36.0±1.6 (<0.001)	3.7±0.7 (<0.001)
** Ly6C**	ND	5.0±1.1 (n.s.)	5.9±0.4	80.3±3.4 (<0.05)	70.6±0.5 (<0.001)

† Percent of positively stained cells for indicated marker was determined by flow cytometry of isolated CD11b^+^ cells collected from either untreated (control) mice or those treated with 50 µg of LPS (n = 20 for each group). Data are presented as the mean percent per group ± SEM.

¥ Statistical significance was determined by Student's t-test comparing the differences between the same populations in control and LPS-treated CD11b-positive cells. Both LPS-P2 and LPS-P3 were compared to Ctrl-P2 due to absence of the third population in the control samples. *P*-values are displayed in parentheses.

§ ND, non-detected.

Although all three LPS-induced populations had increased VEGFR-3 expression, the largest increase (3.37-fold) was associated with LPS-P2 as indicated by the shift from 7.7% in average in control to 26.0% VEGFR-3^+^ cells in LPS-treated mice (Fig. 1E, Table 1). LPS-P2 cells also co-expressed LYVE-1 (8.1%), podoplanin (33.5%), CD11c (36%) and Ly6C (80%) identifying them as monocytic progenitors with pro-lymphatic phenotype. LPS-P1 had similar 2.48-fold increase in VEGFR-3 whereas the new LPS-P3 population contained 5.8% of VEGFR-3^+^ cells. These data indicate that several subsets of CD11b^+^ macrophages upregulate VEGFR-3 on their surface and might promote inflammatory lymphangiogenesis as suggested by their co-expression of lymphatic-specific markers.

### CD11b^+^/VEGFR-3^+^ macrophages isolated from LPS-treated mice display LECP phenotype

To characterize the phenotype of VEGFR-3^+^ macrophages *in vivo*, we isolated CD11b^+^ macrophages from the peritoneal cavity of LPS-treated mice and sorted them by FACS into two populations that either expressed or lacked VEGFR-3. The sorted populations designated as CD11b^+^/VEGFR-3^+^ and CD11b^+^/VEGFR-3^−^ were then analyzed by RT-qPCR to compare the expression of lymphatic- and endothelial-specific markers.

As expected, the level of VEGFR-3 mRNA was 35.6±2.5-fold higher in CD11b^+^/VEGFR-3^+^ cells than in macrophages lacking this receptor (Table 2). Importantly, this cell population was also characterized by increased expression of many other LEC-specific markers, including *cMaf* (2.58±0.51-fold), *CouptfII* (5.22±0.41-fold), *Itga9* (4.50±0.16-fold), *Lyve1* (41.2±3.3), *Neuropilin2* (1.73±0.28), *Notch1* (1.83±0.14), *podoplanin* (4.05±0.18-fold), *Sox17* (4.02±0.09-fold), *Vegfr1* (4.09±0.16), and *Vegfc* (4.41±0.42-fold). Notably, LYVE-1, a major lymphatic cell marker, was robustly elevated by 41-fold in the CD11b^+^/VEGFR-3^+^ subset compared with VEGFR-3^−^ macrophages. Prox1 was a single LEC phenotypic marker that was 2-fold decreased in CD11b^+^/VEGFR-3^+^ cells compared with the negative cells (Table 2). In comparison, several BEC-specific markers were also decreased in this population including *Cd34* (−1.23-fold), *Pecam1* (−1.41-fold), *Tie2* (−1.48-fold) and *Vegfr2* (−2.39-fold). Collectively, these data show that VEGFR-3^+^/CD11b^+^ macrophages display the tendency toward the lymphatic-specific phenotype which is indicated by their relative overexpression of lymphatic-specific proteins and downregulation of BEC-associated proteins. This observation suggests that the lymphatic-specific proteins expressed in this subset may aid in recruitment of LECPs and their integration with lymphatic vessels that subsequently undergo sprouting.

**Table 2:** Differences in gene expression in VEGFR-3^+^ compared with VEGFR-3^−^ macrophages.

Gene	Fold change in gene expression†	*P-*value^‡^	Gene	Fold change in gene expression	*P-*value
Increased			Decreased		
*Bcl3*	1.37±0.17	<0.01	*Akt*	−1.28±0.11	<0.01
*Bclxl*	2.13±0.09	<0.05	*Ang1*	−2.47±0.46	<0.01
*Ccl5*	4.61±0.31	<0.001	*Ang2*	−1.72±0.22	<0.001
*Ccr2*	3.95±0.16	<0.001	*Bcl2*	−9.41±0.66	<0.001
*Ccr5*	2.15±0.03	<0.001	*Ccr1*	−1.29±0.05	<0.01
*cMaf*	2.58±0.51	<0.05	*Ccr3*	−2.08±0.26	<0.001
*CouptfII*	5.22±0.41	<0.001	*Ccr7*	−2.08±0.10	<0.001
*Cox2*	2.71±0.14	<0.001	*Cd34*	−1.23±0.02	n.s.
*Cx3cr1*	2.36±0.13	<0.001	*cKit*	−3.41±0.47	<0.001
*Foxc2*	1.27±0.15	n.s	*Cxcr4*	−1.70±0.18	<0.01
*Il6*	2.27±0.17	<0.05	*Ets1*	−2.17±0.25	<0.001
*Inos*	1.54±0.27	n.s	*Il1β*	−3.06±0.57	<0.01
*Itga9*	4.50±0.16	<0.01	*Ltβ*	−1.80±0.14	<0.05
*Lyve1*	41.20±3.30	<0.001	*nfkb1 (p50)*	−1.40±0.07	<0.001
*mTor*	1.47±0.01	<0.01	*Pecam1*	−1.41±0.26	<0.001
*Neuropillin1*	1.67±0.27	n.s	*Prox1*	−2.30±0.33	<0.001
*Neuropillin2*	1.73±0.28	n.s	*Rela (p65)*	−1.67±0.04	<0.001
*Notch1*	1.83±0.14	<0.05	*Sox18*	−1.82±0.20	<0.001
*Podoplanin*	4.05±0.18	<0.001	*Syk*	−1.95±0.22	<0.01
*Slp76*	1.36±0.09	<0.05	*Tie2*	−1.48±0.05	<0.05
*Sox17*	4.02±0.09	<0.05	*Tlr4*	−1.74±0.18	<0.001
*Sox7*	1.56±0.15	n.s	*Tlr9*	−3.19±0.90	<0.01
*Spred1*	1.69±0.37	n.s	*Tnfα*	−1.52±0.22	<0.001
*Spred2*	1.61±0.12	n.s	*Vegfd*	−6.51±0.51	<0.01
*Tlr2*	2.84±0.16	<0.001	*Vegfr2*	−2.39±0.29	<0.001
*Vegfa*	1.81±0.20	<0.05			
*Vegfc*	4.41±0.42	<0.01			
*Vegfr1*	4.09±0.16	<0.01			
*Vegfr3*	35.60±2.50	<0.001			

† Fold-change in gene expression was determined by RT-qPCR analysis of CD11b^+^/VEGFR-3^+^ and CD11b^+^/VEGFR-3^−^ macrophages from mice treated with 50 µg of LPS for 24 hours. Values are representative of pooled RNA from five independent experiments (total n = 55 mice). Data were normalized per β-actin and presented as the mean fold-change ± SEM.

### LPS-activated CD11b^+^ macrophages are massively recruited to the proximity of lymphatic vessels

**Figure 2:**
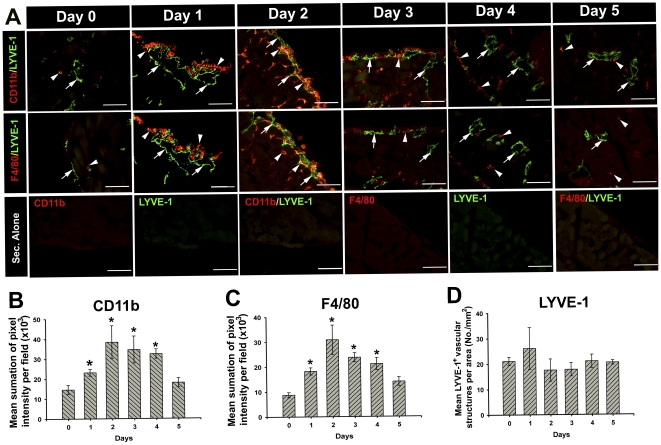
LPS induces recruitment of CD11b^+^ and F4/80^+^ macrophages to the proximity of lymphatic vessels *in vivo*. (**A**) Balb/c mice were injected with 20 µg of LPS for three consecutive days, and sacrificed daily to assess the kinetics of macrophage recruitment to the diaphragm. Control mice represented by Day 0 received 100 µl of sterile, endotoxin-free saline. Diaphragms were co-stained for LYVE-1 and myeloid markers CD11b (panel 1 labeled CD11b/LYVE-1) and F4/80 (panel 2 labeled F4/80/LYVE-1). Secondary controls for each single antibody staining and combinations are presented in the bottom panel labeled “Sec. Alone”. All images were acquired at 200X magnification. White arrows and arrowheads point to LYVE-1 and myeloid markers, respectively. **Note:** integration as indicated by the yellow color mainly occurs on the second day. Sum of pixel intensity per field was calculated as described in the Materials and Methods. (**B**) MFI of CD11b^+^ positive staining ± SEM per field. (**C**) MFI of F4/80^+^ positive staining per field ± SEM. (**D**) Average LYVE-1^+^ structures per mm^2^ of the diaphragm. Statistical significance (*P*<0.05) is denoted by asterisk.

To better understand the behavior of endogenous LECPs, we first analyzed the kinetics of their recruitment into the diaphragm in response to LPS. Group of 3 mice were daily treated with either endotoxin-free saline or 20 µg LPS for three days. Diaphragms were harvested at days 0 to 5 after the first injection and analyzed for co-localization of macrophage markers, CD11b and F4/80, and lymphatic marker, LYVE-1. All secondary IgG controls produced minimal background (Fig. 2A, bottom row). Before LPS treatment, diaphragms contained very few macrophages that were distantly located in relation to the LYVE-1^+^ vessels (Fig. 2A, Day 0). In sharp contrast, 24 hours after LPS treatment, the numbers of tissue-infiltrating macrophages were substantially increased by 3–4 folds in a highly significant statistical manner with *P*-values of 0.04 and 0.006 for CD11b^+^ and F4/80^+^ cells, respectively (Figs. 2B and 2C). These macrophages created large aggregates located in the close proximity to the lymphatic vessels (Fig. 2A, Day 1 and Day 2). The peak of macrophage recruitment was on Day 2 (Fig. 2). On Day 3, the macrophage density was reduced by 20–30% compared with the peak numbers; by Day 5 the density was undistinguishable from normal level in untreated mice (Fig. 2). No change was recorded in LYVE-1^+^ vessel density in the course of first week post-treatment (Fig. 2D) indicating that interactions between pre-existing lymphatic vessels and activated macrophages occur many days prior to genesis of new vessels.

### LPS-activated endogenous macrophages integrate into pre-existing lymphatic vessels *in vivo*

**Figure 3:**
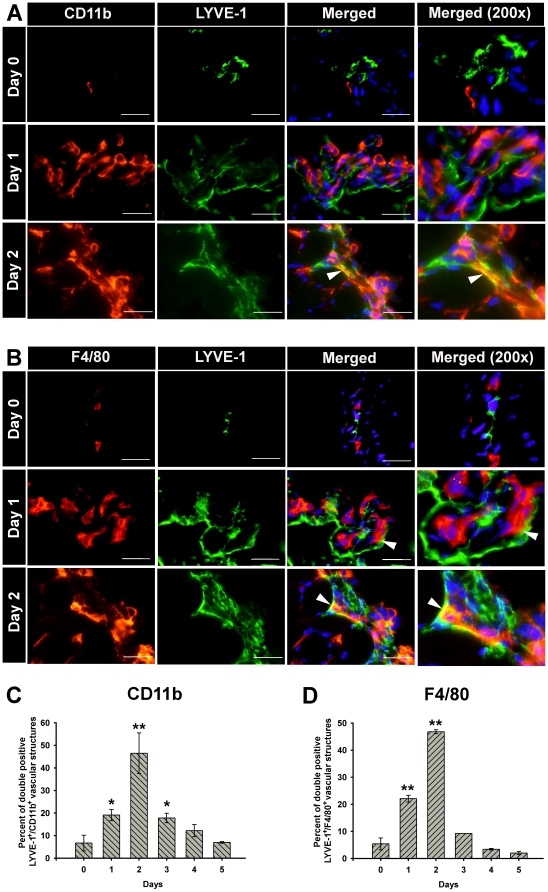
LPS induces integration of endogenous M-LECP into LYVE-1^+^ lymphatic vessels *in vivo*. Diaphragm tissues were co-stained with anti-LYVE-1 and anti-CD11b or F4/80 antibodies. Panels A and B show representative images at days 0, 1, and 2 post-LPS treatment for all markers stained singularly and combined with LYVE-1. All images were acquired using an objective of 60X (total 600X magnification). The forth column in each panel (labeled Merged 200×) shows the merged image magnified 200-fold using Photoshop. On day 0 prior to LPS stimulation, CD11b^+^ (**A**) and F4/80^+^ macrophages (**B**) were completely separated from LYVE-1^+^ vessels. On day 1, subsets of CD11b and F4/80-positive macrophages were found in close proximity to most LYVE-1^+^ vessels. On day 2, CD11b and F4/80 markers were largely co-localized with LYVE-1 indicating integration of endogenous macrophage-derived LECP into pre-existing LYVE-1^+^ lymphatic vessels. White arrowheads indicate co-localization of the two markers. (**C**) The mean percent of LYVE-1^+^ vessels with CD11b^+^ macrophages integrated into the vessels ± SEM. The mean value is derived from analyzing al lymphatic vessel in a diaphragm section for integration of M-LECPs from three individual mice per time point (total n = 9–10). (**D**) Incorporation of F4/80^+^ macrophages was analyzed in a similar manner to that of CD11b^+^ cells. The mean percent of LYVE-1^+^ vessels with integrated F4/80^+^ macrophages ± SEM. Single asterisk and double asterisks indicate statistical significance of a *P*-value<0.01 and <0.001, respectively.

One of the most intriguing and unique properties of LECPs is their ability to integrate into the existing vessels [28], [31]. To compare LECPs from this inflammatory model with those described previously [28], [31], we determined the extent and the temporal pattern of incorporation of CD11b^+^ and F4/80^+^ macrophages into pre-existing lymphatic vasculature. Fig. 3 shows representative serial sections from LPS-treated mice at day 0, 1 or 2 post-treatment that were double-stained with anti-LYVE-1 and macrophage markers. We found that while recruitment to the periendothelial lymphatic space and attachment to the LEC surface occur mainly at the first day (Figs. 3A and 3B, Day 1), the majority of integration events mainly occur at Day 2 post-treatment in comparison with other days (Figs. 3C and 3D).

At the peak of integration phase, Day 2, nearly 50% of LYVE-1^+^ vessels co-expressed CD11b and F4/80 as indicated by the yellow color on the merged images (Figs. 3A and 3B). The differences in frequencies of co-localization between days 1–3 and other days of the study were highly statistically significant with *P*-values ranging between 0.045 and 0.002.

The integration phase identified by dual expression of the lymphatic and myeloid markers began to slow down on the third day of treatment reaching undetectable level on the fifth day (Figs. 3C and 3D). This suggests that after the macrophages incorporate into the lymphatic vessels, they lost the myeloid markers while overexpressing the lymphatic markers making them indistinguishable from the recipient cells. As shown on serial sections in Figs. 3A and 3B, both the frequency and the extent of integration of F4/80^+^ macrophages were nearly identical to those of CD11b^+^ cells. This observation in consistent with the evidence for VEGFR-3 upregulation in both resident and recruited LPS-activated macrophages (Table 1), thus suggesting that both mature and immature macrophages are sufficiently plastic to structurally contribute to growing lymphatic vessels.

### LPS-activated RAW264.7 macrophages *in vitro* display *do novo* expression of VEGFR-3

Characterization of *in vivo* LPS-activated macrophages revealed significant increase in VEGFR-3 expression in up to 26% of CD11b^+^ cells (Fig. 1, Table 1) concomitant with upregulation of many other LEC-specific genes (Table 2). We hypothesized that these events can be modeled *in vitro* using a macrophage cell line RAW264.7 [42] activated by LPS. The rationale to create a new *in vitro*, system modeling macrophage-LEC differentiation process was the expected ability to perform in-depth molecular analyses typically not achievable *in vivo* due to complexity at the whole animal level.

**Figure 4:**
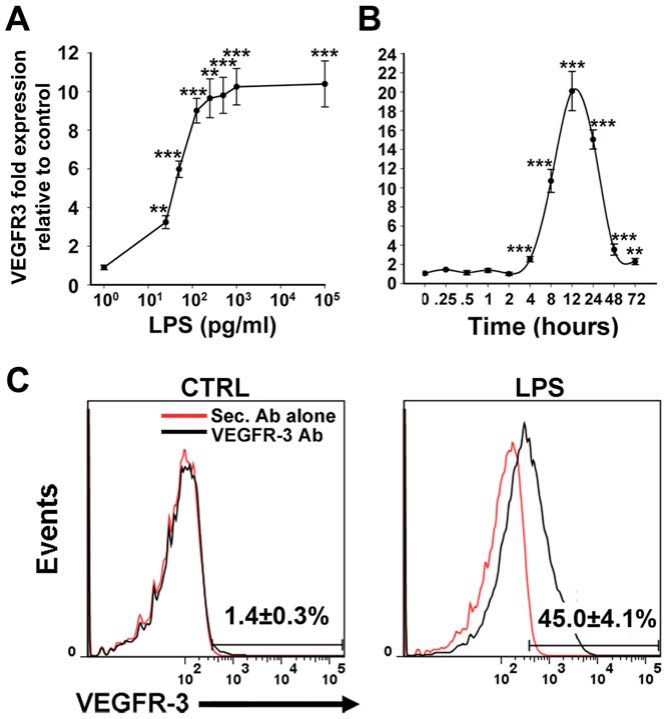
LPS upregulates VEGFR-3 mRNA and protein in cultured RAW264.7 macrophages in a dose- and time-dependent manner. (**A**) VEGFR-3 mRNA in RAW264.7 macrophages treated with LPS (0–100 ng/ml) for 24 hours was analyzed by RT-qPCR and compared with untreated controls (n = 6 per group). (**B**) RT-qPCR analysis of VEGFR-3 mRNA in RAW264.7 macrophages treated with 100 ng/ml LPS for 0 to 72 hours (n = 9 per group). For (**A**) and (**B**), relative transcript expression was normalized to β-actin. Data are presented as β-actin normalized transcript expression ± SEM. The *P*-values represent **<0.01 and ***<0.001 versus control as determined by Student's unpaired *t-*test. (**C**) Flow cytometry analysis of VEGFR-3 protein expression on the surface of RAW264.7 macrophages that were treated with 100 ng/ml of LPS for 24 hours. Values represent the percentage of VEGFR-3^+^ macrophages from 3 independent experiments performed in triplicate ± SEM (total n = 9 per group).

As a proof-of-principle for establishing such a system, we characterized the sensitivity and the kinetics of inflammation-induced VEGFR-3 expression in LPS-treated RAW264.7 macrophages *in vitro*. To measure the sensitivity of VEGFR-3 induction, cells were activated with 0–100 ng/ml of LPS for 24 hours followed by RT-qPCR analysis. VEGFR-3 expression increased by 3.2±0.3-fold in response to as little as 0.025 ng/ml of LPS followed by linear upregulation to 9.7-fold at 0.5 ng/ml of LPS, with no further increase above this dose (Fig. 4A). To characterize the kinetics of VEGFR-3 expression, cells were exposed to 100 ng/ml of LPS for 0–72 hours followed by RT-qPCR analysis. Compared with control, VEGFR-3 expression increased by 2.5±0.3-fold 4 hours post-exposure, peaked to 20±2-fold at 12 hours, and remained elevated by 2.3±0.3-fold at 72 hours post-treatment (Fig. 4B). To determine whether mRNA correlated with increased VEGFR-3 cell-surface protein, RAW264.7 macrophages were activated by 100 ng/ml of LPS for 24 hours and analyzed by flow cytometry. Fig. 4C shows that LPS treatment increased cell-surface VEGFR-3 protein by 32-fold (*P*<0.05) from 1.4±0.3% in control macrophages to 45.0±4.1% in LPS-activated cell population.

Collectively, these data show that LPS at picogram concentrations induces significant changes in VEGFR-3 mRNA and protein expression in both macrophage-derived LECPs *in vivo* and RAW264.7 macrophages *in vitro*. These changes are characterized by a rapid peak at 12 hours of a 20-fold increase in mRNA followed by a 32-fold increase in surface-presented VEGFR-3 protein 24 hours post-exposure. The rapidity of this response, the precise timing of mRNA upregulation, and the substantial increase in this protein on cell surface all suggest an important regulatory role of VEGFR-3 in early stages of macrophage differentiation into LECPs.

### *De novo* expression of VEGFR-3 expression is preceded by activation of NF-κB

**Figure 5:**
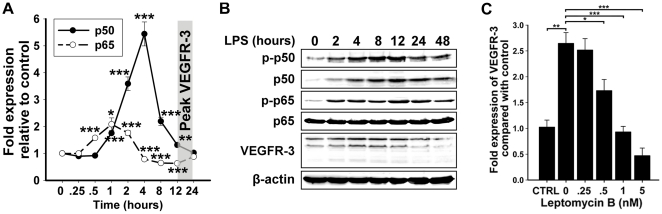
Activation of NF-κB signaling precedes elevation of VEGFR-3 in LPS-treated RAW264.7 macrophages. (**A**) RT-qPCR analysis of NF-κB p50 and p65 expression in RAW264.7 macrophages treated with 100 ng/ml LPS for 0–24 hours (n = 6 per group). Relative transcript expression was normalized to β-actin. Data presented as β-actin normalized transcript expression ± SEM. The *P*-values represent *<0.05, **<0.01, and ***<0.001 versus control as determined by Student's unpaired *t* test. (**B**) Protein expression of NF-κB p50 phosphorylated on Ser-337 (phospho-p50), non-phosphorylated NF-κB p50, NF-κB p65 phosphorylated on Ser-276 (phospho-p65), non-phosphorylated NF-κB p65, VEGFR-3, and β-actin was determined by Western blot in RAW264.7 macrophages treated with 100 ng/ml of LPS for 0–48 hours. Representative blot from two independent experiments performed in triplicate wells is shown (total n = 6 per time point). (**C**) RT-qPCR analysis of LPS-induced VEGFR-3 expression in the presence of NF-κB inhibitor, leptomycin B. Relative transcript expression was normalized to β-actin. Data presented as β-actin normalized transcript expression ± SEM. CTRL (taken as 1) represents the mean basal VEGFR-3 expression in untreated RAW264.7 macrophages. The *P*-values represent *<0.05, **<0.01, and ***<0.001 *versus* control as determined by Student's unpaired *t* test.

We previously reported that p50 and p65 subunits of NF-κB regulate VEGFR-3 expression on LECs *in vitro* and in lymphatic vessels during inflammation *in vivo* [17], [43]. We hypothesized that LPS-induced VEGFR-3 in macrophage-derived LECPs might also be regulated by NF-κB. To test this hypothesis, we compared the expression of NF-κB p50, p65, and VEGFR-3 in RAW264.7 macrophages treated with 100 ng/ml of LPS or PBS for 0–24 hours. Four hours after LPS treatment, transcripts of NF-κB p50 and p65 increased respectively by 5.4±0.4 and 2.1±0.2-fold (Fig. 5A). This was paralleled by an increase in total and phosphorylated NF-κB p50 protein as well as phosphorylated NF-κB p65 although non-phosphorylated p65 protein level remained unchanged (Fig. 5B). Importantly, phosphorylation of NF-κB proteins preceded VEGFR-3 upregulation by 4–8 hours (Fig. 5B) suggesting that functionally-active NF-κB regulates VEGFR-3 on the transcriptional level. We previously showed that VEGFR-3 expression depends on activation of NF-κB by using an NF-κB inhibitor, leptomycin B [17]. Consistent with our prior findings [17], treatment of RAW264.7 macrophages with leptomycin B (10 nM) inhibited VEGFR-3 response to LPS in a dose-dependent manner (Fig. 5C). Collectively, these data suggest that NF-κB regulates transcription of VEGFR-3 in both LECs and macrophage-derived LECPs.

### LPS-treated RAW264.7 macrophages activate autocrine VEGF-C•VEGFR-3 loop

**Figure 6:**
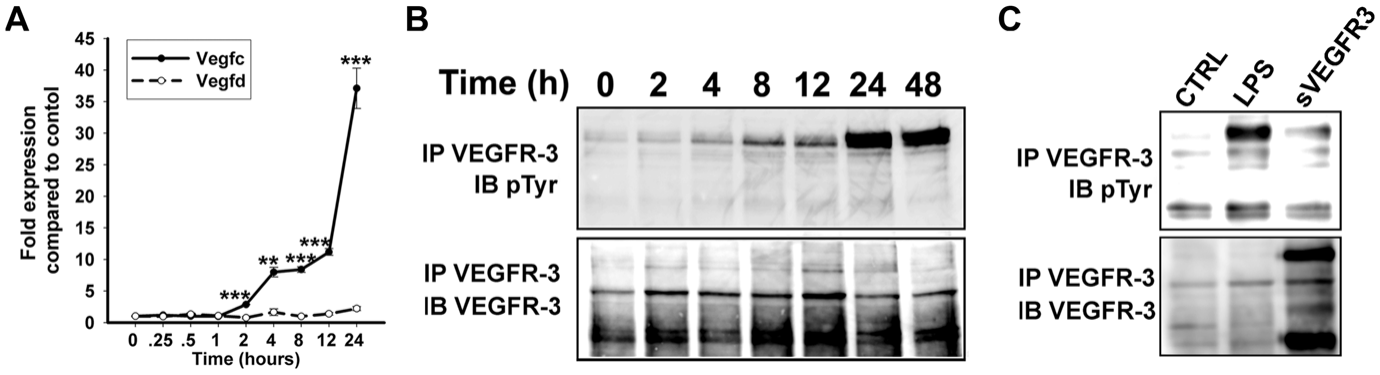
LPS induces autocrine VEGFR-3 phosphorylation in RAW264.7 macrophages *in vitro*. (**A**) RT-qPCR analysis of VEGF-C and VEGF-D mRNA expression in RAW264.7 macrophages treated with 100 ng/ml of LPS for 0–24 hours. The relative expression was normalized to β-actin. Data are presented as the mean values performed in triplicate ± SEM (total n = 3). The *P*-values represent **<0.01 and ***<0.001 *versus* control as determined by Student's unpaired *t* test. (**B**) VEGFR-3 protein was immunoprecipitated using anti-VEGFR-3 antibodies from whole cell lysates of RAW264.7 macrophages treated with 100 ng/ml of LPS for 0–48 hours. Immunoprecipitated proteins were blotted and probed with anti-pTyr and anti-VEGFR-3 antibodies to determine phosphorylation status of VEGFR-3. Representative blot from two independent experiments performed in triplicate is shown (total n = 6 per timepoint). (**C**) RAW264.7 macrophages treated with 100 ng/ml of LPS for 24 hours in the presence of soluble VEGFR-3-Fc or irrelevant antibody. VEGFR-3 protein was immunoprecipitated with anti-VEGFR-3 antibody and receptor phosphorylation was analyzed by Western blot using anti-p-Tyrosine antibody. As a loading control, immunoprecipitated VEGFR-3 protein was re-blotted using anti-VEGFR-3 antibodies. Representative image is shown from one experiment performed in triplicate wells (n = 3).

Although NF-κB involvement suggested similarities between macrophages and LEC with respect to regulation of VEGFR-3 expression, the major difference between these cell types is the absence of detectable VEGFR-3 in resting macrophages or undifferentiated myeloid progenitors prior to activation. Our data show that activated macrophages display unique pattern of *de novo* VEGFR-3 expression: the response is transient, potent and fast. This suggests that VEGFR-3 play a unique regulatory role in transdifferentiation of macrophages into lymphatic progenitors. We hypothesized that as a prerequisite for executing this role, VEGFR-3 signaling must be induced in an autocrine manner. This hypothesis was supported by the well-recognized ability of inflammation-activated macrophages to overexpress VEGFR-3 ligands, VEGF-C and VEGF-D [39]. To test this hypothesis, we first quantified expression levels of VEGF-C and VEGF-D in RAW264.7 macrophages treated with 100 ng/ml of LPS for 0 to 24 hours. In contrast to some reports [44], [45], LPS-activated macrophages did not upregulated VEGF-D (Fig. 6A). In sharp contrast, VEGF-C expression doubled after 2 hours of LPS treatment followed by an exponentially increasing level up to nearly 40-fold increase a day later (Fig. 6A).

Consistent with our hypothesis, this finding suggested that simultaneous elevation of VEGFR-3 and VEGF-C may activate autocrine signaling. To test for this possibility, LPS-treated RAW264.7 macrophages (0–48 hours) were analyzed for tyrosine phosphorylation on VEGFR-3 receptor by using immunoprecipitation and Western blot to detect phosphotyrosine. As shown in Fig. 6B, LPS linearly increased phosphorylation of VEGFR-3 up to 14-fold in a time-dependent manner, whereas soluble VEGFR-3-Fc fusion protein completely blocked this event (Fig. 6C). Because VEGFR-3-Fc specifically blocks VEGF-C-dependent activation [46], these data present clear evidence for an inflammation-induced VEGFR-3 autocrine loop, thus further underscoring the functional significance of *de novo* expression of VEGFR-3 for macrophage differentiation into lymphatic progenitors.

### Autocrine VEGFR-3 signaling is followed by upregulation of multitude of lymphatic genes mimicking the profile of endogenous LECPs

The transient kinetic profile of VEGFR-3 expression (Fig. 4) followed by induction of the autocrine loop (Fig. 6) suggested that VEGFR-3 signaling at the early stages of macrophage transformation to LECPs might be essential for switching to the lymphatic phenotype. To test this hypothesis, we analyzed the same set of lymphatic-specific or -associated genes as was performed for VEGFR-3-positive macrophage-derived LECPs *in vivo* (Table 2). RAW264.7 cells were stimulated by 100 ng/ml of LPS for 5 or 24 hours followed by RT-qPCR analysis. 

**Figure 7:**
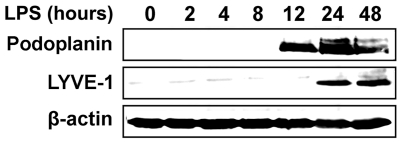
LPS-treated RAW264.7 macrophages acquire lymphatic-specific gene expression. Western blot analysis of Prox1, podoplanin, and LYVE-1 proteins in RAW264.7 macrophages treated with 100 ng/ml of LPS for 0–48 hours. Representative blot from two independent experiments performed in triplicate wells is shown (total n = 6 per timepoint).

Compared with control macrophages, LPS-activated cells significantly increased expression of several LEC markers including VEGFR-3, VEGF-C, LYVE-1, Notch1, alpha integrin 9, c-Maf, and podoplanin. Moreover, phenotypic BEC markers (e.g., CD34, Tie2, VEGFR-2 and neuropilin-1) were coincidently downregulated (Table S1) suggesting a shift toward the lymphatic phenotype. Furthermore, comparison of LPS-activated RAW264.7 with CD11b^+^/VEGFR-3^+^ macrophages isolated from an *in vivo* showed a 68% overlap in gene expression of 37 out of 54 examined genes. VEGFR-3, LYVE-1 and podoplanin were among highly upregulated genes, as evidenced by 12±0.2-fold, 17.1±0.45-fold, and 5,621±89-fold increase in their mRNA expression in LPS-treated RAW264.7 cells (Table S1). Importantly, as demonstrated in Figs. 6 and 7, the corresponding proteins for these genes were absent in untreated macrophages and during early stages of differentiation, but abundantly present after 10 to 12 hours. This *de novo* expression of exclusive lymphatic markers in activated macrophages suggests that they play important roles in defining the lymphatic identity in nascent progenitors and preparing them for lymphatic vascular integration.

### *In vitro* generated GFP-tagged RAW264.7-derived LECPs integrate into lymphatic vessels *in vivo*

The ultimate test for generating experimental LECPs is their demonstrable ability to incorporate into inflamed lymphatic vessels *in vivo*. To determine the capacity of LPS-activated RAW264.7 cells to perform this essential LECP function, we first engineered a sub-line that stably expresses GFP. As described in detail in the Methods, several monoclonal GFP-labeled RAW264.7 line derivatives with unaltered morphology and identical rates of proliferation and LPS response were combined to create a GFP-tagged sub-line designated as RAW-GFP. This sub-line was further taken for *in vivo* analyses designed to determine biodistribution and vascular integration of RAW264.7 cell-derived LECPs in saline-treated (control) and LPS-treated mice.

**Figure 8:**
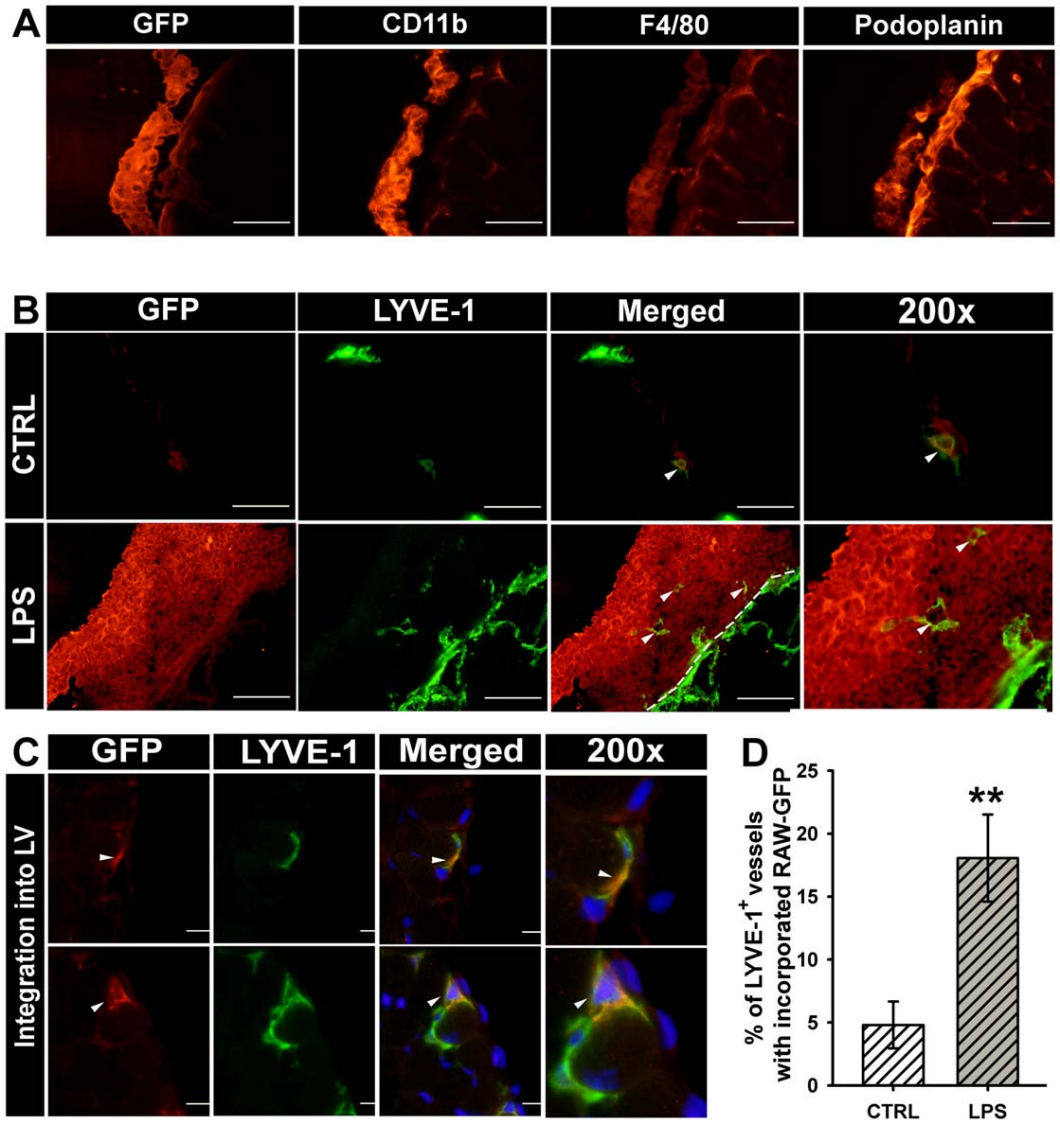
GFP tagged RAW264.7 cells (RAW-GFP) are recruited to LPS-treated diaphragms and undergo lymphatic vascular integration *in vivo*. Balb/c mice were pre-treated with endotoxin-free saline or 20 µg of LPS for three consecutive days prior to i.p. injection of 2×10^6^ RAW-GFP cells. Seven days later, diaphragms were collected and analyzed for triple expression of GFP, lymphatic and myeloid markers. (**A**) Characterization of RAW-GFP cells *in vivo*. Cells maintained the expression of GFP (panel 1), CD11b (panel 2), weak F4/80 (panel 3), and novel expression of a lymphatic-specific marker, podoplanin (panel 4). All images were acquired at 400X magnification. (**B**) Recruitment of RAW-GFP to inflamed, but not control, lymphatic vessels in the diaphragms of saline-treated mice or LPS. Upper panel: RAW-GFP cells were barely detected as a few isolated cells in diaphragms of saline-treated mice. Bottom panel: Massive clusters of tightly adhered RAW-GFP were detected alongside of all peripheral lymphatic vessels in the diaphragms of LPS-treated mice. Representative images from the groups of 3–4 mice are shown. All images were acquired at 200X magnification. (**C**) RAW-GFP cells identified by anti-GFP specific antibody (red structures detected by DyLight 549-conjugated secondary IgG) were found to be fully integrated into LYVE-1^+^ vessels (green structures identified by FITC-conjugated secondary IgG to anti-LYVE-1). Integration of RAW-GFP is clearly indicated by the yellow color on merged (acquired at 600X magnification) and 200-fold amplified merged images (last column).

For the vascular integration assay, two groups of mice (n = 5 per group) were pre-treated with either sterile endotoxin-free saline or 20 µg LPS for three days. The mice were then injected with 2×10^6^ RAW-GFP cells, and sacrificed seven days after cell injection. The presence and location of RAW-GFP cells in the collected diaphragms was determined by staining with anti-GFP followed by DyLight 549 conjugated secondary antibody that generated red color. Because the fluorescent intensity of DyLight 549 is much stronger than other conjugated dyes, we found that this method of GFP-tagged cells is more reliable than staining with green fluorescence emitting dyes or relying on the natural GFP fluorescence. Using this method, we first determined on parallel sections of diaphragms from LPS-treated mice that RAW-GFP cells continued to strongly express not only GFP but also myeloid (e.g., CD11b) and lymphatic (e.g., podoplanin) markers under *in vivo* conditions (Fig. 8A). In contrast, F4/80, a late differentiation myeloid marker [40], was only weakly detected in these cells (Fig. 8A). At this time point (i.e., a week after injection) RAW-GFP cells were negative for VEGFR-3, which is in line with the transient nature of VEGFR-3 upregulation reported in this study (Fig. 4).

Next, we determined whether RAW-GFP behave differently in control and LPS-treated mice. Double immunostaining for LYVE-1 and GFP identified very few recruited cells to the control diaphragm (Fig. 8B, upper row), and a small percentage (4.8%) of control lymphatic vessels to be associated with injected cells (Fig. 8, B–D).In contrast, both density and the extent of vascular incorporation of RAW-GFP in LPS-treated mice surpassed those in the control mice by several folds. In contrast to the control group, all mice in the LPS-treated group showed massive recruitment of the RAW-GFP cells to the inflamed diaphragm, with many humongous clusters strongly attached to the peritoneal side of the diaphragm (Fig. 8B, bottom row). Moreover, LYVE-1-positive vascular tube-like structures were frequently observed within the RAW-GFP aggregates (white arrows in Fig. 8B, merged and 200X). In addition to these macrophage-autonomously generated lymphatic vessels outside of the tissue, RAW-GFP cells also incorporated into the lymphatic vessels within the inflamed diaphragm. The dual LYVE-1 and GFP staining identified approximately 18% of the lymphatic vessels with coincident expression of GFP (Fig. 8C). The extent of lymphatic vascular integration was 3.75-fold higher in LPS-treated mice than in control mice, the difference that was highly statistically significant with a *P*-value of less than 0.001 (Fig. 8D). These data indicate that LPS-activated RAW264.7 cells not only can model macrophage-lymphatic transition *in vitro* but also reproduce the LECP behavior *in vivo*.

## Discussion

LECPs are defined as circulating BM-derived cells with *de novo* expression of lymphatic markers and the capacity to integrate into growing lymphatic vessels. Although LECPs have been observed in human tissues [29], [47], [48] and animal models [28], [30], [31], [35], the progress in understanding their biology is currently impeded by low frequency *in vivo* [27], [28], [30], inability to follow their fate due to post-integration loss of myeloid markers, and most importantly, lack of representative cell culture models. We sought to establish an *in vitro* model reproducing the LECP phenotype that could be easily manipulated and robustly analyzed at the molecular, cellular, and whole animal levels.

To this end, we used an immortalized macrophage cell line RAW264.7 [42] that lacks LEC markers under normal conditions. We found that LPS treatment of this line *in vitro* induces coincident expression of VEGFR-3 and VEGF-C, which creates a positive autocrine loop. This activation of VEGFR-3 signaling appears to be a prerequisite for induction of a broad panel of lymphatic-specific genes that are similarly upregulated in both endogenous M-LECPs and LPS-stimulated RAW264.7 cells *in vitro* (Table 2 and Table S1). The LPS-activated RAW264.7 cells appear to recapitulate several fundamental features of endogenous LECPs including potent but transient expression of VEGFR-3, upregulation of a diverse panel of lymphatic-specific markers, massive infiltration into inflamed tissue, firm attachment to activated lymphatic endothelium, and finally, widespread incorporation into lymphatic vessels. These characteristics of LECPs found in the peritonitis model (Figs. 1, 2, 3 and Tables 1–2) have been previously identified in LECPs detected in a variety of other inflammatory models [30], [33]. Having access to a culture model that mimics the basic features of the M-LECPs partaking in inflammatory lymphangiogenesis *in vivo* should allow us to dissect the molecular details of this process.

### LPS induces the lymphatic phenotype in endogenous macrophages *in vivo*

Prior to establishing the cell culture model, we sought to characterize the endogenous M-LECPs in a mouse peritonitis model that has been previously used to demonstrate induction of LPS- and macrophage-dependent inflammatory lymphangiogenesis [11], [17]. We first focused on peritoneal macrophages that have the potential to become LECPs. To our knowledge, such analysis has not been done previously, although it can facilitate understanding of the mechanisms regulating LECP recruitment, infiltration into inflamed tissue, and subsequent integration into vessels.

Using this model, we found that control mice had two distinct populations of resident peritoneal macrophages whereas LPS-treated mice had three. Importantly, all three LPS-induced populations (LPS-P1, -P2 and -P3) had statistically significant increases in VEGFR-3 expression (Fig. 1 and Table 1). The largest increase was observed in LPS-P2, a subset that also expressed LYVE-1, podoplanin, and a set of markers often found in BM-recruited monocyte progenitors (i.e., CD11b^+^/F480^−^/CD11c^+^/Ly6C^high^). The other two subsets (LPS-P1 and LPS-P3) contain 6–9% of VEGFR-3^+^ cells that might also be part of the LECP pool. The LPS-P1 and -P3 subsets were characterized, respectively, by the CD11b^+^/F480^high^/CD11c^−^/Ly6C^−^ and CD11b^+^/F480^low^/CD11c^low^/Ly6C^high^ profiles, and might represent an activated resident and a recruited progenitor sub-population distinct from LPS-P2. It, therefore, stands to reason that M-LECPs in the peritonitis model might originate from at least three different macrophage sub-populations: two major ones with high Ly6C expression recruited from the bone marrow, and one minor subset characterized by F480^high^ that might be derived from the resident macrophages activated *in situ*.

### VEGFR-3 might play a critical role in the early phase of M-LECP differentiation

Although VEGFR-3 was highly expressed on up to 26–28% in some populations of activated macrophages, this marker was nearly undetectable on diaphragm-infiltrated macrophages after the first day of treatment (Fig. S1). Analogously, LPS-treated RAW264.7 cells *in vitro* showed a sharp bell-shaped pattern of VEGFR-3 upregulation peaking at 12 hours and returning to the basal levels after 48 hours (Fig. 4B). These similarities, in both the potency and the transient nature of the expression, suggest that VEGFR-3 plays a critical regulatory role at the early phase of macrophage-to-LECP differentiation, but might not be required for fulfilling later LECP functions. Studies on the RAW264.7 cells also showed that co-expression of VEGFR-3 with VEGF-C generate an autocrine loop (Fig. 6). It is, therefore, tempting to propose that transient activation of VEGFR-3 autocrine signaling may restrict the lymphatic path to selected subsets of progenitors that undergo further pro-lymphatic differentiation by transcribing lymphatic-specific genes that ultimately allow LECP integration into lymphatic vasculature.

This concept is consistent with the substantial increase in a variety of *de novo* transcribed lymphatic-specific or -associated genes in both LPS-activated endogenous CD11b^+^/VEGFR-3^+^ macrophages (Table 2) and RAW264.7 macrophages *in vitro* (Table S1). In addition to VEGFR-3, we also found high upregulation of LYVE-1, podoplanin, COUP-TFII, Sox7, Notch1 and alpha 9 integrin. These genes upregulated in CD11b^+^/VEGFR-3^+^ macrophages play critical roles in the formation of embryonic lymphatic vasculature. For instance, the pro-lymphatic roles of Sox7 and Notch1 have been shown to regulate VEGFR-3 in embryonic LECPs [49]–[51]. Likewise, genetic ablation of endothelial COUP-TFII disrupts formation of the lymphatic system due to inability of venous-derived LECPs to fully differentiate into mature LECs [52], [53]. LYVE-1 [54], podoplanin [55], and integrin alpha9 [56] are all well-known specific markers of LECs as well as their precursors [27], [31]. These findings are, therefore, consistent with the idea that LPS causes the lymphatic differentiation in macrophages and monocyte progenitors by forcing *de novo* expression of genes with critical roles in embryonic lymphangiogenesis. This observation suggests that expression of these genes in postnatal inflammation-activated macrophages regulate LECP differentiation, and subsequently, support their function in the newly-established lymphatic vessels.

Among all markers examined, two lymphatic endothelial specific proteins, Prox1 and Tie2, were downregulated in VEGFR-3^+^ macrophages in both *in vivo* and *in vitro* assays. Prox1 has been detected in embryonic [32] and MSC-derived [35] LECPs but not in adult LECPs originated from the myeloid lineage. Taken collectively with our data, it might imply that generation of M-LECP does not require Prox1 expression. Tie2 has been reported to be expressed in a subset of myeloid pro-angiogenic progenitors [57], [58], and, therefore, was a good candidate for a marker of macrophage-derived precursors with lymphangiogenic properties. However, both Tie2 mRNA (Table 2 and Table S1) and surface-expressed protein (not shown) were found to be either downregulated or undetected in VEGFR-3^+^ macrophages compared with controls. Tie2 expressing monocytes (TEMs) have been reported to express F480^+^/CD11c^−^/Ly-6C^−^/LYVE-1^+^ and implicated in promotion of tumor angiogenesis [59]. In contrast, the subpopulation of M-LECPs we identified were F480^−^/CD11c^+^/Ly-6C^+^/LYVE-1^+^ indicating a separate subset of monocytes progenitors that are actively involved in inflammatory lymphangiogensis (Fig. 3). Future studies in the LPS-driven RAW264.7 model may clarify questions regarding potential roles of Prox1 and Tie2 in inflammation-induced M-LECPs.

### RAW264.7 macrophages treated by LPS *in vitro* acquire the lymphatic phenotype

Analysis of LPS-activated cultured RAW264.7 macrophages revealed significant upregulation of a broad panel of lymphatic-specific genes consistent with *de novo* acquisition of the lymphatic phenotype (Table S1). Increased at both mRNA and protein levels (Table S1, Figs. 4, 5 & 7), these markers encompassed many known pro-lymphangiogenic genes including VEGFR-3, podoplanin, integrin alpha9, Notch1, and LYVE-1. Sixty-eight percent of these genes were similarly detected in endogenous CD11b^+^/VEGFR-3^+^ macrophages (Table 2) and in inflammation-induced LECPs identified in independent studies [29], [31], [33], [60]. These data suggest that LPS-activated RAW264.7 macrophages *in vitro* acquire the essential features phenotypic of LECPs detected at lymphangiogenic sites *in vivo*.

One of the earliest upregulated genes in both RAW264.7 cells and endogenous M-LECPs was VEGFR-3. Both endogenous and RAW264.7 LPS-treated macrophages shared similarities in the pattern of the VEGFR-3 response to the LPS characterized by high sensitivity, rapidity and transient nature (Fig. 4). The sensitivity of VEGFR-3 induction suggests a specific response mediated by an LPS receptor, TLR4, rather than general response to stress. This is also suggested by dependence of VEGFR-3 induction on NF-κB in both activated M-LECPs (Fig. 5) and differentiated LECs responding to inflammatory stimuli [17]. However, in contrast to adult LECs, VEGFR-3 expression in macrophages and macrophage-derived progenitors returned to the basal levels within 48 hours (Fig. 4B). This suggests that VEGFR-3 is only necessary to set up the initial stage for differentiation but is not required for maintaining the lymphatic identity.

Treatment with LPS upregulated both VEGFR-3 and VEGF-C expressions thus creating a novel autocrine loop (Fig. 6). The expression of VEGF-C by activated macrophages was previously proposed to be important for chemotaxis of VEGFR-3^+^ macrophages [37], integration of BM-derived VEGFR-3^+^ LECPs into lymphatic vessels [30], and induction of VEGFR-3 signaling in differentiated LEC [8], [44], [45]. Our data suggest that in addition to these functions, VEGF-C is also needed for activation of the autocrine VEGFR-3 signaling that leads to transcription of LECP differentiation genes. This hypothesis is consistent with studies showing that VEGF-C treatment induces lymphatic differentiation in progenitor cells such as ESC [61] and BMDC [31]. Moreover, differentiation of ESC-derived LECPs has been shown to be blocked by forced expression of either mutated [34] or a soluble VEGFR-3 receptor [61]. These studies and our findings collectively suggest that early activation of VEGFR-3•VEGF-C axis in macrophages during inflammation might be necessary for ensuring the lymphatic identity in progenitor cells as well as their incorporation into lymphatic vessels.

The ultimate test for the ability of RAW264.7 cells to function as LECPs is demonstration of their recruitment and integration lymphatic vessels *in vivo*. We show here that GFP-tagged RAW264.7 cells (RAW-GFP) indeed have the capacity to mimic the behavior of endogenous M-LECPs in both recruitment to and incorporation into inflamed lymphatic vessels. Like the endogenous M-LECP that intimately associated with the lymphatic endothelium prior to integration (Fig. 3), large aggregates of RAW-GFP were detected in the proximity of the lymphatic vessels in LPS-treated mice (Fig. 8). Moreover, 18% of the lymphatic vessels co-expressed GFP and LYVE-1 indicating a significant ability of experimentally generated M-LECPs to functionally perform as native lymphatic precursors by structurally contributing to growing lymphatic vasculature.

Collectively, these data show that LPS-activated RAW264.7 cells not only phenocopy the lymphatic gene profile of endogenous M-LECPs but also their functional capacity to integrate into lymphatic vasculature.

### The RAW264.7 model of macrophage-LECP differentiation offers numerous advantages for studying lymphatic biology *in vivo*

Our primary goal was to establish a cell-based model that would allow in-depth characterization of macrophage differentiation into LECPs observed during inflammatory lymphangiogenesis *in vivo*. To this end, we selected a macrophage line RAW264.7 that faithfully recapitulates the macrophage phenotype [62], [63] and is exquisitely sensitive to inflammatory stimuli. As such, it provides a solid platform for studying macrophage transition to LECPs induced by an inflammatory trigger such as LPS.

We showed that LPS-activated RAW264.7 macrophages display the lymphatic-specific gene signature largely overlaps with that of endogenous CD11b^+^/VEGFR-3^+^ LECPs (Table S1). Many of the genes upregulated in LECPs in response to inflammation (e.g., podoplanin, integrin alpha9, Notch1, COUP-TFII, and Sox7) have been implicated in embryonic lymphatic development, yet a similar function in adult has not been established. The RAW264.7 macrophage model can be used for defining the LECP-specific functions of these genes through techniques, such as fluorescent labeling, gene knockdown, and transgene overexpression. This line can easily be manipulated *in vitro* to change the expression of these genes following by *in vivo* transplantation into genetically compatible Balb/c mice [42]. We, therefore, propose that this system designated for simplicity as “the RAW model” can be used for in-depth analysis of the molecular mechanisms regulating LECP functions in postnatal lymphangiogenesis, a currently understudied field due to low frequency of endogenous LECPs and the complexity of dissecting multifaceted processes *in vivo*. We anticipate that the RAW model described here will help to overcome current challenges in the field thus opening the door for exciting new analyses of M-LECPs leading to a better understanding of the lymphatic biology.

## Materials and Methods

### Ethics Statement

The animal experiments were carried out in strict accordance with the recommendations in the Guide for the Care and Use of Laboratory Animals of the National Institute of Health. The protocol was approved by the Laboratory Animal Care and Use Committee of the Southern Illinois University School of Medicine (protocol number 187-11-007).

### Materials

LPS derived from *Escherichia coli 055:*B5, TRI-Reagent, endotoxin-free sterile saline, protease inhibitor cocktail, and PMSF were purchased from Sigma-Aldrich (St. Louis, MO). Dulbecco's modified Eagle's medium (DMEM), Dulbecco phosphate buffered saline (DPBS), and all standard medium supplements were from Lonza (Basel, Switzerland). Mouse anesthetic cocktail consisted of ketamine (Fort Dodge Animal Health, Fort Dodge, Iowa), xylazine (Phoenix Scientific Inc., St. Joseph, MO) and sterile water.

### Antibodies

We used the following primary antibodies: rat anti-mLYVE-1, goat anti-mVEGFR-3, -mLYVE-1, and -GFP (R&D Systems, Minneapolis, MN); hamster anti-mPodoplanin, rabbit anti-mLYVE-1 and anti-Prox1 (AngioBio, Del Mar, CA); mouse anti-phospho-tyrosine (p-Tyr), rabbit anti-p65, anti-p50, and anti-phospo-p50 (Santa Cruz, Santa Cruz, CA); rabbit anti-phospo-p65 (Epitomics, Burlingame, CA); mouse anti-β-actin, clone JLA20 (Developmental Studies Hybridoma Bank, Iowa City, IA); rat anti-F4/80 (Invitrogen, Carlsbad, CA); PE-conjugated rat anti-mCD11b and anti-Ly6C; FITC-conjugated rat anti-CD11c, and rat anti-Ly6G/C (Becton-Dickinson, Franklin Lakes, NJ); and biotinylated rat anti-mTie2 (eBioscience, San Diego, CA). Secondary antibodies conjugated to HRP, FITC, DyLight 488, DyLight 549, and APC conjugated to donkey anti-rabbit and anti-goat IgG, streptavidin and non-specific rabbit antibodies were all from Jackson ImmunoResearch Laboratories (West Grove, PA).

### Mouse model of LPS-induced peritonitis

Balb/c female mice (4–6 weeks) were obtained from Harlan Laboratory (Indianapolis, IN) and treated in accordance with institutional guidelines. Control mice were injected with 200 µl sterile endotoxin-free saline. Peritonitis was induced by repetitive i.p. injections on days 0, 1, and 2 with 20 µg of LPS dissolved in 200 µl of sterile endotoxin-free saline. On days 0, 1, 2, 3, 4 and 5 of the study, mice were anesthetized by a ketamine/xylazine cocktail and perfused with 5 mM CaCl_2_ solution. Diaphragms were collected from perfused mice and snap-frozen immediately.

### Fluorescence-activated cell sorting (FACS) and analyses of LPS-recruited CD11b^+^/VEGFR-3^−^ and CD11b^+^/VEGFR-3^+^ macrophages

Four independent experiments were performed using Balb/c female mice injected i.p. with 50 µg of LPS diluted in 100 µl of sterile endotoxin-free saline (total n = 50). Activated peritoneal macrophages were collected 24 hours post-injection by lavage using 10 ml of cold DMEM with 10% FBS and double-stained with goat anti-mVEGFR-3 and rat anti-mCD11b antibodies using the following protocol. Cells were centrifuged at 100 RCF for 5 minutes and resuspended at a density of 1×10^6^ cells per 100 µl of F-buffer (PBS containing 2.5% horse serum) supplemented with 4 µg/ml of non-specific mouse IgG (Sigma, St. Louis, MO). After 15-minute incubation on ice to block non-specific binding to Fc receptors, cells were washed several times and resuspended in 100 µl of F-buffer containing 4 µg/ml of goat anti-mVEGFR-3 antibody. After a 30-minute incubation on ice, cells were washed three times in FACS buffer and resuspended in 100 µl of the F-buffer containing 5 µg/ml of APC-conjugated donkey anti-goat antibody and 2 µg/ml of PE-conjugated rat anti-mCD11b antibodies. After additional 30-minute incubation on ice, cells were washed again in F-buffer. Following immunostaining, CD11b^+^/VEGFR-3^−^ and CD11b^+^/VEGFR-3^+^ cell populations were isolated using a FACSAriaII high-speed cell sorter (Becton-Dickinson, Franklin Lakes, NJ). Based on FACS analysis, the purity of CD11b^+^ cells was greater than 95%. After sorting, RNA was extracted from CD11b^+^/VEGFR-3^−^ and CD11b^+^/VEGFR-3^+^ cell populations by TRI-Reagent, according to the manufacturer's protocol, and gene expression was analyzed by RT-qPCR as described below.

### Preparation of LPS-recruited peritoneal macrophages for flow cytometry analysis

Mice were treated and peritoneal macrophages were harvested by lavage (n = 5–6 mice per group per experiment) as stated above. CD11b^+^ cells were isolated using rat anti-mCD11b magnetic beads (Miltenyi Biotec, Auburn, CA) and fixed with 1% paraformaldehyde for 15 minutes on ice. Fixed cells were double-stained with goat anti-mVEGFR-3 and rat anti-mCD11b, -mLYVE-1, -Ly6C, -F4/80, -CD11c, -mTie2, or hamster anti-mPodoplanin antibodies as described above. Marker expression was analyzed by flow cytometry using Accuri C6 flow cytometer (BD Accuri Cytometers, Ann Arbor, MI). Similar procedure was used to analyze expression of VEGFR-3 on cultured RAW264.7 macrophages treated with 100 ng/ml LPS or DPBS (control) for 24 hours. All analyses were reproduced in at least three independent experiments.

### Immunofluorescent staining

All antibodies were diluted 1∶100 in PBST (pH 7.4, 0.1% Tween-20) containing 5 µg/ml of BSA. Frozen sections were fixed with acetone for 10 minutes, rehydrated in PBST for 10 minutes and incubated for 1 hour at 37°C with primary antibodies against macrophage markers (CD11b or F4/80) and mouse LYVE-1. Slides were washed and incubated for 1 hour at 37°C with DyLight 488- or 549-conjugated secondary antibodies. For double immunofluorescent staining, sections were incubated with primary and secondary antibodies at 37°C for 1 hour, respectively, with a 10 minute wash in PBST between steps. Slides were mounted in Vectashield medium containing 4,6′-diamidino-2-phenylindole (DAPI) nuclear stain (Vector Labs, Orton Southgate, U.K.). Images were acquired using an Olympus BX41 upright microscope equipped with a DP70 digital camera and DP Controller software (Olympus, Center Valley, PA).

### Measurement of mean fluorescent intensity

The mean fluorescent intensity (MFI) of CD11b and F4/80 positive staining was calculated as described previously [17], with slight modifications. Briefly, fluorescent images were acquired at a constant exposure time at 200X magnification on an Olympus BX41 upright microscope equipped with a DP70 digital camera and DP Controller. To exclude background staining, sections stained with secondary antibodies only were used to set the exposure time to the level below background fluorescence. Digital RGB images acquired at a constant exposure time were converted to 8-bit grayscale. The fluorescent intensity for each pixel was calculated using the histogram function of Image J (http://rsbweb.nih.gov/ij/) that was set up in the linear intensity range of 0 to 255 arbitrary units. Using this scale, background fluorescent intensity of tissues stained with secondary antibodies alone was less than 10 units. MFI was calculated as the mean of the total pixels above background in four images per slide derived from individual mice in each group (n = 3 per group). The results are presented as averaged MFI arbitrary units per group ± SEM.

### Quantification of LYVE-1^+^ vessel density

Frozen sections of diaphragms were acetone-fixed for 10 minutes and stained with antibody against the lymphatic-specific marker, LYVE-1, for 1 hour at 37°C, followed by incubation with DyLight 488-conjugated donkey anti-rabbit secondary antibodies for 1 hour at 37°C. To quantify LYVE-1 positive vessel density, all LYVE-1^+^ structures in the diaphragm section were enumerated. The total area of the diaphragm was then measured using Image J software. LYVE-1 counts were then normalized per mm^2^ of diaphragm area. Lymphatic vessel density is presented as the normalized number of LYVE-1^+^ vessels per area of the field ± SEM (n = 3–4 mice per group).

### Quantification of M-LECP incorporation into LYVE-1^+^ vessels

Diaphragms were double-stained for CD11b or F4/80 and LYVE-1 antibodies. Each LYVE-1^+^ vessel in the diaphragm section was individually assessed for co-localization with anti-CD11b and anti-F4/80 antibodies using different filters in Olympus BX41 microscope. The percentage of incorporated vessels was calculated by dividing the number of LYVE-1^+^vessel with incorporated macrophages by total number of LYVE-1^+^ vessels in the diaphragm section. The results are presented as the mean percent of vessels with incorporated M-LECP ± SEM derived from 3 mice per group.

### RT-qPCR analysis

Two micrograms of total RNA was reverse transcribed using a RevertAid First Strand cDNA synthesis kit, according to the manufacturer's protocol (Fermentas, Burlington, Ontario, Canada). Primers for RT-qPCR were designed against mouse and human CDS of angiogenic and lymphangiogenic proteins found in the NCBI database. Primer sequences were chosen using the Harvard primer database website (http://pga.mgh.harvard.edu/primerbank/index.html) and validated for specificity and primer efficiency. All primers (listed in supplementary Table S2) were purchased as annealed oligos from Integrated DNA Technologies (Coralville, IA). Quantitative RT-PCR was performed using GoTaq qPCR Master Mix (Promega, Madison, WI) and either an ABI 7500 Real-Time (Applied BioSystems, Foster City, CA) or a Mastercycler ep *realplex* (Eppendorf, Hamburg, Germany) PCR machine. A typical reaction consisted of an initial denaturation step at 95°C for 5 minutes followed by 40 cycles of denaturation at 95°C for 15 seconds, and annealing, extension, and data acquisition at 60°C for 1 minute. A final melting curve for each primer was calculated by heating from 60°C to 90°C. Data were normalized to β-actin and relative mRNA expression was determined using the ΔΔCt method described previously [35], [64].

### Immunoprecipitation and Western blot analysis

The mouse RAW264.7 macrophage cell line (ATCC, Manassas, VA), was cultured in DMEM supplemented with 10% FBS and standard additives. For analysis of VEGFR-3 phosphorylation, RAW264.7 macrophages were treated with 100 ng/ml of LPS for 0–48 hours. In some experiments, RAW264.7 macrophages were pre-treated for 2 hours with soluble VEGFR-3-Fc recombinant protein (3 µg/ml; R&D Systems, Minneapolis, MN), followed by stimulation with 1 ng/ml of LPS for 24 hours. Following treatment, cells were washed twice with ice-cold DPBS, lysed in ice-cold lysis buffer (50 mM Tris-HCl, pH 7.5, 150 mM NaCl, 1 mM EDTA, 1% Triton-X100, 0.1% SDS, 200 mM PMSF, protease inhibitors and phosphatase inhibitor cocktails), and spun down for 10 minutes at 13,000 RCF to remove insoluble material. Protein concentration was determined by Bradford assay, and 750 µg of lysate protein was incubated with 2 µg of goat anti-mVEGFR-3 antibodies (R&D Systems, Minneapolis, MN) for 12 hours at 4°C. VEGFR-3 protein-antibody complexes were precipitated by incubating with 30 µl of magnetic beads conjugated to protein G (4 hours at 4°C). Beads were washed thrice in ice-cold lysis buffer and VEGFR-3-antibody complexes were eluted by boiling for 10 minutes in 50 µl of Laemmli buffer containing 10 mM DTT. Eluted proteins were separated in a 9% SDS-PAGE gel, transferred to nitrocellulose membranes that were blocked with 5% milk in PBST for 1 hour, and incubated overnight at 4°C with anti-pTyr antibody. Membranes were washed thrice with PBST, followed by 1 hour incubation at room-temperature with HRP-conjugated secondary antibodies. After additional washing in PBST, membranes were developed with ECL (Pierce, Rockford, IL) for 5 minutes. Protein bands were visualized using a Fujifilm LAS-3000 camera and analyzed with Image-Reader LAS-3000 software (Valhalla, NY). To visualize the total amount of VEGFR-3 pull-down, membranes were stripped with buffer consisting of 1.5% glycine (w/v), 0.1% SDS, 1% Tween-20, pH 2.2, following by re-probing with anti-VEGFR-3 and HRP-conjugated secondary antibodies.

To analyze the kinetics of NF-κB and LEC-specific protein expression, RAW264.7 macrophages were treated with 100 ng/ml of LPS for 0–48 hours and analyzed by Western blot as described above. Protein lysates were separated on 9–12% SDS-PAGE gels and probed overnight at 4°C with antibodies against Prox1, VEGFR-3, podoplanin and LYVE-1, followed by 1 hour incubation with species-appropriate HRP-conjugated secondary antibodies. Protein bands were detected as described above and densitometric analysis was performed using Image J software.

### Generation of RAW264.7 cells tagged with Green Fluorescent Protein (GFP)

RAW264.7 cells were seeded at a density of 200,000 cells per well in a 6-well plate with 2 ml of DMEM. Cells were allowed to adhere to the plate overnight. Cells were washed with serum-free DMEM for 20 minutes before adding supernatant containing a GFP-encoding lentivirus with SFFV promoter (a generous gift from Dr. Wilber, SIU School of Medicine). Medium was refreshed 24 hours later and GFP-expressing cells detected by direct microscopy in the subsequent week were isolated using FACS. The enriched population was then sub-cloned by a limiting dilution in a 96-well plate. Cells derived from monoclonal colonies with homogeneous GFP expression and parental morphology were selected for further analyzes. Clones were expanded and tested for identical and unaltered proliferation rate as well as LPS response as compared with the unmodified RAW264.7 cell line. Several of these clones were combined to create a GFP-tagged RAW264.7 sub-line designated here as RAW-GFP.

### RAW-GFP macrophage incorporation into pre-existing LYVE-1^+^ vessels

Balb/c female mice were injected with 200 µl of sterile endotoxin-free saline (control group) or 20 µg of LPS for three consecutive days. After stimulation, mice were injected with 2×10^6^ of untreated RAW-GFP cells i.p. After 7 days, mice were sacrificed; the diaphragms were harvested and analyzed by immunofluorescence for expression of GFP and co-localization of GFP with myeloid and lymphatic markers.

### Statistical analysis

Statistical analysis was performed using SAS software (SAS Institute, Inc., Cary, NC). All results are expressed as the mean ± SEM and statistical differences were assessed by unpaired Student's *t*-test. Statistical significance was defined as *P*<0.05.

## Supporting Information

Figure S1 **CD11b^+^ macrophages are recruited to VEGFR-3^+^ vessels**. Balb/c mice were injected with 20 µg of LPS once, and sacrificed daily thereafter to determine whether the recruited macrophages express VEGFR-3. Diaphragms were co-stained for VEGFR-3 and CD11b (upper panel). LYVE-1^+^ vessels recruited CD11b-posiitve macrophages but these macrophages were largely negative for VEGFR-3. Secondary controls for each single antibody staining and combinations are presented in the lower panel. All images were acquired at 200X magnification.

Table S1 **Relative change in gene expression profile of LPS-activated vs. untreated RAW264.7 macrophages.**
(DOCX)

Table S2 **Sequences of primers used for qRT-PCR.**
(DOCX)
