# Supplementary figures and images for: New Model of Macrophage Acquisition of the Lymphatic Endothelial Phenotype
Source: PLoS One. 2012 Mar 2;7(3):e31794. doi: 10.1371/journal.pone.0031794 (PMC3292559; doi:10.1371/journal.pone.0031794)

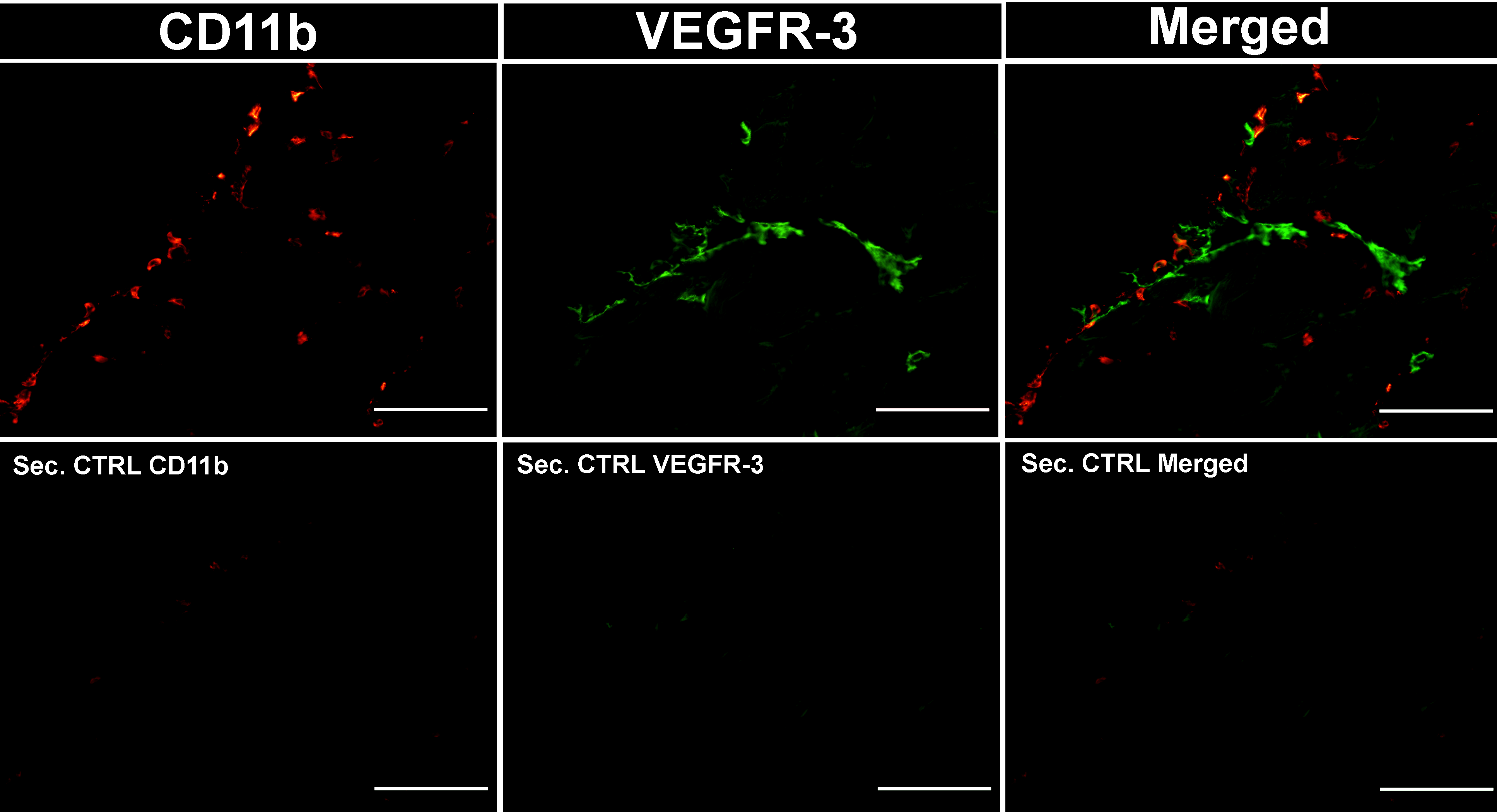

Supplement: Figure S1 — CD11b+ macrophages are recruited to VEGFR-3+ vessels. Balb/c mice were injected with 20 µg of LPS once, and sacrificed daily thereafter to determine whether the recruited macrophages express VEGFR-3. Diaphragms were co-stained for VEGFR-3 and CD11b (upper panel). LYVE-1+ vessels recruited CD11b-posiitve macrophages but these macrophages were largely negative for VEGFR-3. Secondary controls for each single antibody staining and combinations are presented in the lower panel. All images were acquired at 200X magnification. [file pone.0031794.s001.tif]
