# Supplementary material for: New Model of Macrophage Acquisition of the Lymphatic Endothelial Phenotype
Source: PLoS One. 2012 Mar 2;7(3):e31794. doi: 10.1371/journal.pone.0031794 (PMC3292559; doi:10.1371/journal.pone.0031794)
Supplement: Table S1 — Relative change in gene expression profile of LPS-activated vs. untreated RAW264.7 macrophages. (DOCX) [file pone.0031794.s002.docx]

**Table S1.** Relative change in gene expression profile of LPS-activated vs. untreated RAW264.7 macrophages

|  | **5h LPS vs. control** | | **24h LPS vs. control** | | |
| --- | --- | --- | --- | --- | --- |
|  | **Fold-change^†^** | ***P-*value^‡^** | **Fold-change** | ***P-*value** | |
| **Increased** | | | | | |
| *Bcl3* | 6.63 ± 0.09 | <0.001 | 3.93 ± 0.34 | | <0.001 |
| *Bclxl* | 4.64 ± 0.30 | <0.001 | 4.81 ± 0.27 | | <0.001 |
| *Ccl5* | 1,822.00 ± 172.00 | <0.01 | 1,552.00 ± 108.00 | | <0.01 |
| *Ccr1* | 1.70 ± 0.05 | n.s. | 185.00 ± 16.00 | | <0.01 |
| *Ccr3* | 4.68 ± 0.01 | <0.05 | 31.30 ± 1.30 | | <0.001 |
| *Ccr7* | 4.66 ± 0.22 | <0.05 | 31.60 ± 1.70 | | <0.001 |
| *cMaf* | 260.00 ± 19.00 | <0.01 | 2,210.00 ± 318.00 | | <0.05 |
| *Cox2* | 81.50 ± 5.40 | <0.01 | 133.00 ± 9.90 | | n.s. |
| *Il6* | 41,979.00 ± 5,574.00 | <0.05 | 117,973.00 ± 12,390.00 | | <0.05 |
| *Inos* | 664.00 ± 53.00 | <0.01 | 6,990.00 ± 302.00 | | <0.01 |
| *Itga9* | 8.99 ± 0.46 | <0.05 | 11.70 ± 1.50 | | <0.05 |
| *Ltβ* | 1.55 ± 0.06 | n.s. | -13.80 ± 0.44 | | <0.001 |
| *Lyve1* | 17.01 ± 0.45 | <0.001 | 10.00 ± 0.78 | | <0.01 |
| *nfkb1 (p50)* | 3.78 ± 0.13 | <0.001 | 1.19 ± 0.07 | | n.s. |
| *Notch1* | 5.68 ± 0.27 | <0.001 | 3.13 ± 0.19 | | <0.001 |
| *Pecam1* | 2.90 ± 0.40 | <0.05 | 9.14 ± 0.31 | | <0.001 |
| *Podoplanin* | 54.70 ± 3.20 | <0.01 | 5,621.00 ± 89.00 | | <0.001 |
| *Slp76* | 1.37 ± 0.30 | n.s. | 2.20 ± 0.05 | | 0.001 |
| *Sox17* | 1.59 ± 0.07 | n.s. | -2.72 ± 0.25 | | n.s. |
| *Syk* | 3.32 ± 0.05 | <0.01 | 6.13 ± 0.43 | | <0.001 |
| *Tlr2* | 1.38 ± 0.09 | n.s. | -4.73 ± 0.49 | | 0.01 |
| *Tnfα* | 20.03 ± 1.30 | <0.01 | 3.38 ± 0.10 | | 0.01 |
| *Vegfa* | 3.80 ± 0.04 | <0.001 | 6.99 ± 0.48 | | <0.001 |
| *Vegfc* | 15.82 ± 1.00 | <0.001 | 409.00 ± 21.00 | | <0.001 |
| *Vegfr3* | 2.39 ± 0.15 | <0.01 | 12.00 ± 0.80 | | <0.001 |
| ***Decreased*** | | | | | |
| *Akt* | -3.67 ± 0.22 | <0.001 | -3.80 ± 0.63 | | <0.05 |
| *Ang1* | -31.50 ± 2.90 | n.s. | -4.50 ± 1.2 | | <0.05 |
| *Ang2* | -3.42 ± 0.83 | n.s. | -4.17 ± 0.98 | | n.s. |
| *Bcl2* | -25.20 ± 5.00 | <0.05 | -5.63 ± 0.97 | | <0.05 |
| *Ccr2* | -1.85 ± 0.08 | n.s. | -1.18 ± 0.07 | | n.s. |
| *Ccr5* | -3.19 ± 0.39 | <0.05 | 1.42 ± 0.16 | | n.s. |
| *Cd34* | -9.16 ± 0.52 | <0.01 | -6.45 ± 0.15 | | <0.05 |
| *cKit* | -6.57 ± 1.00 | <0.05 | -5.03 ± 0.60 | | <0.05 |
| *CouptfII* | -4.74 ± 0.64 | n.s. | -5.90 ± 0.77 | | <0.05 |
| *Cx3cr1* | -15.30 ± 0.51 | <0.001 | -122.00 ± 8.2 | | <0.001 |
| *Cxcr4* | -35.90 ± 3.80 | 0.05 | -3.62 ± 1.10 | | n.s. |
| *Ets1* | -1.82 ± 0.07 | <0.01 | 2.56 ± 0.18 | | <0.01 |
| *Foxc2* | -8.52 ± 0.45 | <0.01 | -4.82 ± 0.79 | | <0.05 |
| *Il1β* | -1.78 ± 0.22 | n.s. | -2.08 ± 0.19 | | n.s. |
| *mTor* | -2.04 ± 0.24 | <0.05 | -3.05 ± 0.38 | | <0.01 |
| *Neuropillin1* | -8.12 ± 0.39 | <0.01 | -2.44 ± 0.41 | | <0.05 |
| *Neuropillin2* | -1.35 ± 0.12 | <0.05 | 1.52 ± 0.13 | | <0.05 |
| *Prox1* | -3.30 ± 0.49 | n.s. | -11.40 ± 0.86 | | <0.01 |
| *Rela (p65)* | -1.46 ± 0.19 | n.s. | -1.30 ± 0.05 | | <0.05 |
| *Sox18* | -2.11 ± 0.13 | <0.01 | -3.51 ± 0.37 | | <0.01 |
| *Sox7* | -3.63 ± 0.47 | <0.05 | -2.19 ± 0.39 | | <0.05 |
| *Spred1* | -3.08 ± 0.17 | n.s. | -2.75 ± 0.28 | | n.s. |
| *Spred2* | -12.60 ± 1.20 | <0.01 | -1.97 ± 0.19 | | <0.05 |
| *Tie2* | -4.85 ± 0.33 | 0.001 | -3.34 ± 0.40 | | <0.01 |
| *Tlr4* | -5.03 ± 0.38 | 0.01 | 1.06 ± 0.21 | | n.s. |
| *Tlr9* | -2.78 ± 0.48 | <0.05 | -3.99 ± 0.23 | | <0.001 |
| *Vegfd* | -4.77 ± 0.09 | n.s. | -5.22 ± 0.07 | | n.s. |
| *Vegfr1* | -1.97 ± 0.08 | 0.001 | -1.90 ± 0.03 | | 0.001 |
| *Vegfr2* | -1.53 ± 0.11 | n.s. | -1.24 ± 0.10 | | n.s. |

**^†^**Fold-change in gene expression was determined by RT-qPCR analysis of macrophages treated with LPS (100 ng/ml) compared with untreated control macrophages. Β-actin normalized pooled data from 3 to 4 independent experiments per group are presented as the mean fold-change ± SEM.

^‡^*P*-value was determined by Student’s unpaired t-test.

^§^n.s., non-significant changes
