# Supplementary material for: New Model of Macrophage Acquisition of the Lymphatic Endothelial Phenotype
Source: PLoS One. 2012 Mar 2;7(3):e31794. doi: 10.1371/journal.pone.0031794 (PMC3292559; doi:10.1371/journal.pone.0031794)
Supplement: Table S2 — Sequences of primers used for qRT-PCR. (DOCX) [file pone.0031794.s003.docx]

**Table S2.** Sequences of primers used for qRT-PCR

| Gene | Forward primer sequence | Reverse primer sequence |
| --- | --- | --- |
| Akt | 5'-ATGAACGACGTAGCCATTGTG-3' | 5'-TTGTAGCCAATAAAGGTGCCAT-3' |
| Ang1 | 5'-CACATAGGGTGCAGCAACCA-3' | 5'-CGTCGTGTTCTGGAAGAATGA-3' |
| Ang2 | 5'-TGGTACTACTGGAAGGGGTCCG-3' | 5'-GAAATCTGCTGGCCGGATCA-3' |
| β-actin | 5'-GGCTGTATTCCCCTCCATCG-3' | 5'-CCAGTTGGTAACAATGCCATGT-3' |
| Bcl2 | 5'-ATGCCTTTGTGGAACTATATGGC-3' | 5'-GGTATGCACCCAGAGTGATGC-3' |
| Bcl3 | 5'-ACTCACCCCTACTCCATGATATG-3' | 5'-GCGGCTATGTTATTCTGGACCA-3' |
| Bclxl | 5'-GACAAGGAGATGCAGGTATTGG-3' | 5'-TCCCGTAGAGATCCACAAAAGT-3' |
| Ccl5 | 5'-GCTGCTTTGCCTACCTCTCC-3' | 5'-TCGAGTGACAAACACGACTGC-3' |
| Ccr1 | 5'-CTCATGCAGCATAGGAGGCTT-3' | 5'-ACATGGCATCACCAAAAATCCA-3' |
| Ccr2 | 5'-ATCCACGGCATACTATCAACATC-3' | 5'-CAAGGCTCACCATCATCGTAG-3' |
| Ccr3 | 5'-TCAACTTGGCAATTTCTGACCT-3' | 5'-CAGCATGGACGATAGCCAGG-3' |
| Ccr5 | 5'-TTTTCAAGGGTCAGTTCCGAC-3' | 5'-GGAAGACCATCATGTTACCCAC-3' |
| Ccr7 | 5'-TGTACGAGTCGGTGTGCTTC-3' | 5'-GGTAGGTATCCGTCATGGTCTTG-3' |
| Cd34 | 5'-AAGGCTGGGTGAAGACCCTTA-3' | 5'-TGAATGGCCGTTTCTGGAAGT-3' |
| cMaf | 5'-TGTCTCTGCTGCACCCTCTTG-3' | 5'-AGCAAGGAGGAGGTGATCCG-3' |
| CoupTFII | 5'-TTCACCCATGTCAGCCGAC-3' | 5'-GGCCTTGAGGCAGCTATACTC-3' |
| Cox2 | 5'-TGAGCAACTATTCCAAACCAGC-3' | 5'-GCACGTAGTCTTCGATCACTATC-3' |
| Cx3cr1 | 5'-GAGTATGACGATTCTGCTGAGG-3' | 5'-CAGACCGAACGTGAAGACGAG-3' |
| Cxcr4 | 5'-GAAGTGGGGTCTGGAGACTAT-3' | 5'-TTGCCGACTATGCCAGTCAAG-3' |
| Ets1 | 5'-CGGGTCCCCTCCTATGACAG-3' | 5'-GAATGACAGGCTTGTCCTTGTT-3' |
| Foxc2 | 5'-AACCCAACAGCAAACTTTCCC-3' | 5'-GCGTAGCTCGATAGGGCAG-3' |
| Il1β | 5'-GCAACTGTTCCTGAACTCAACT-3' | 5'-ATCTTTTGGGGTCCGTCAACT-3' |
| Il6 | 5'-TAGTCCTTCCTACCCCAATTTCC-3' | 5'-TTGGTCCTTAGCCACTCCTTC-3' |
| Inos | 5'-GTTCTCAGCCCAACAATACAAGA-3' | 5'-GTGGACGGGTCGATGTCAC-3' |
| Itga9 | 5'-AAGTGTCGTGTCCATACCAAC-3' | 5'-GGTCTGCTTCGTAGTAGATGTTC-3' |
| Ltβ | 5'-TGGCAGGAGCTACTTCCCT-3' | 5'-TCCAGTCTTTTCTGAGCCTGT-3' |
| Lyve1 | 5'-CAGCACACTAGCCTGGTGTTA-3' | 5'-CGCCCATGATTCTGCATGTAGA-3' |
| Mmp9 | 5'-GGACCCGAAGCGGACATTG-3' | 5'-CGTCGTCGAAATGGGCATCT-3' |
| mTor | 5'-ACCGGCACACATTTGAAGAAG-3' | 5'-CTCGTTGAGGATCAGCAAGG-3' |
| Neuropillin-1 | 5'-GACAAATGTGGCGGGACCATA-3' | 5'-TGGATTAGCCATTCACACTTCTC-3' |
| Neuropillin-2 | 5'-GCTGGCTACATCACTTCCCC-3' | 5'-CAATCCACTCACAGTTCTGGTG-3' |
| Nfkb1 (p50) | 5'-CAAACTGTCTGTGAACATCCG-3' | 5'-AAATGGTGGAGTTTGGGAAGGAT-3' |
| Notch1 | 5'-GCCGCAAGAGGCTTGAGAT-3' | 5'-GGAGTCCTGGCATCGTTGG-3' |
| Pecam1 | 5'-CTGGTGCTCTATGCAAGCCTC-3' | 5'-CGGTGCTGAGACCTGCTTT-3' |
| Podoplanin | 5'-ACCGTGCCAGTGTTGTTCTG-3' | 5'-AGCACCTGTGGTTGTTATTTTGT-3' |
| Prox1 | 5'-TACCAGGTCTACGACAGCACCG-3' | 5'-GTCTTCAGACAGGTCGCCATC-3' |
| Rela (p65) | 5'-GCTACACGGGACCAGGAACAG-3' | 5'-AGTTCATGTGGATGAGGCCG-3' |
| Slp76 | 5'-AGAGGACTTCCTGTCTGTATCAG-3' | 5'-TGGACCCTCGATTCTTTCCATC-3' |
| Sox17 | 5'-GATGCGGGATACGCCAGTG-3' | 5'-CCACCACCTCGCCTTTCAC-3' |
| Sox18 | 5'-CGCAGGTCTCTACTATGGCAC-3' | 5'-TAGTGGCATCCGGTCGAGT-3' |
| Sox7 | 5'-AGAACACGCTGCCTGAGAAAA-3' | 5'-TCCTGACATGAGGACGAGAAG-3' |
| Spred1 | 5'-GAGATGACTCAAGTGGTGGATG-3' | 5'-TCTGAAAGGTAAGGCCAAACTTC-3' |
| Spred2 | 5'-GAAGAAACACACCCGGACGAT-3' | 5'-CTCCTTCCTGTGGGAACCAT-3' |
| Syk | 5'-CTACCTGCTACGCCAGAGC-3' | 5'-GCCATTAAGTTCCCTCTCGATG-3' |
| Tie2 | 5'-CGGCCAGGTACATAGGAGGAA-3' | 5'-TCACATCTCCGAACAATCAGC-3' |
| Tlr2 | 5'-GCAAACGCTGTTCTGCTCAG-3' | 5'-AGGCGTCTCCCTCTATTGTATT-3' |
| Tlr4 | 5'-ATGGCATGGCTTACACCACC-3' | 5'-GAGGCCAATTTTGTCTCCACA-3' |
| Tlr9 | 5'-ATGGTTCTCCGTCGAAGGACT-3' | 5'-GAGGCTTCAGCTCACAGGG-3' |
| Tnfa | 5'-CCCTCACACTCAGATCATCTTCT-3' | 5'-GCTACGACGTGGGCTACAG-3' |
| Vegfa | 5'-GCACATAGAGAGAATGAGCTTCC-3' | 5'-CTCCGCTCTGAACAAGGCT-3' |
| Vegfc | 5'-GAGGTCAAGGCTTTTGAAGGC-3' | 5'-CTGTCCTGGTATTGAGGGTGG-3' |
| Vegfd | 5'-GAGGACTGGAAGCTGTGGCG-3' | 5'-GCGTGAGTCCATACTGGCAAGA-3' |
| Vegfr1 | 5'-CTCAGACAAGTCAAACCTGGAG-3' | 5'-GGGAACTTCATCTGGGTCCATAA-3' |
| Vegfr2 | 5'-TACCCACTTGTCTGCCCTGGT-3' | 5'-TCCTGTCTGAGCCTTTACCG-3' |
| Vegfr3 | 5'-CTGGCAAATGGTTACTCCATGA-3' | 5'-ACAACCCGTGTGTCTTCACTG-3' |
